# Children of the grave: Investigating non-adult feeding practices in medieval and early modern Estonia through stable isotope analysis

**DOI:** 10.1371/journal.pone.0279546

**Published:** 2023-01-04

**Authors:** Alessandra Morrone, Mari Tõrv, Dario Piombino-Mascali, Tina Saupe, Holar Sepp, Heiki Valk, Martin Malve, Ester Oras

**Affiliations:** 1 Department of Archaeology, Institute of History and Archaeology, Faculty of Arts and Humanities, University of Tartu, Tartu, Estonia; 2 Department of Analytical Chemistry, Institute of Chemistry, Faculty of Science and Technology, University of Tartu, Tartu, Estonia; 3 Department of Anatomy, Histology, and Anthropology, Institute of Biomedical Sciences, Faculty of Medicine, Vilnius University, Vilnius, Lithuania; 4 PalaeoMole Group, Estonian Biocentre, Institute of Genomics, Faculty of Science and Technology, University of Tartu, Tartu, Estonia; 5 Department of Geology, Institute of Ecology and Earth Sciences, Faculty of Science and Technology, University of Tartu, Tartu, Estonia; University of Padova: Universita degli Studi di Padova, ITALY

## Abstract

Studying infant diet and feeding practices through stable isotope analysis provides direct insight into the life and health of vulnerable population groups in the past. Although the general diet in medieval and early modern Livonia has been reconstructed from written sources, little is known about childhood diet during this tumultuous period of Eastern European history. This study presents a comparative investigation of the staple non-adult diet in urban/rural communities during the 13^th^-17^th^ centuries AD, with a special focus on feeding practices. We aim to reveal the impact of socio-economic circumstances on early childhood nutrition, which affects the physical development and overall survival of this susceptible population group. Bone collagen samples from 176 individuals between the fetal and the 7–15 age categories from four urban/rural South-Estonian cemeteries were cross-sectionally analyzed via EA-IRMS (Elemental Analysis with Isotope Ratio Mass Spectroscopy) for δ^13^C and δ^15^N. Results suggest that South-Estonian children had a staple terrestrial C_3_ diet integrated with animal proteins. Significant divergences were observed between urban and rural sites and slight variation occurred among rural subgroups, possibly resulting from a wider food choice available in towns, different consumption of C_4_ foods, and/or secular changes. This study provides the first data regarding infant feeding practices in medieval and early modern Livonia. These practices were similar among the different contexts, indicating comparable cultural traditions in child rearing. Breastfeeding was likely practiced for 1–2 years, with supplementary foods introduced around 1 year of age. The weaning process was probably concluded around the age of 3. The δ^13^C and δ^15^N values of older children are comparable to those of the adults from the same sites, indicating their diets became similar after weaning, when they started working and obtained a more mature social status.

## 1. Introduction

The investigation of infant diet and feeding practices allows for the reconstruction of fundamental aspects of everyday life in archaeological populations. From a socio-cultural point of view, understanding these practices helps reveal the complex interactions between childcare in different socio-economic contexts, treatment of minorities, and local traditions in dietary selection [1, 2]. On the biological side, understanding these practices offers valuable insights into population demography, nutrition, and disease patterns of mothers and offspring [2–4]. Breastfeeding habits are deeply intertwined within the cultural framework of societies, within family traditions, and among each mother-child nexus, also constituting means for family planning and self-regulation for well-being and social status of fertile women [3–6]. The duration of breastfeeding and the subsequent initiation of the weaning process (*i*.*e*. the introduction of supplementary foods, alongside with breastmilk) is therefore the result of a set of complex interactions, often affecting the ability of the individuals to cope with nutritional and physiological stress [7–10]. The introduction of complementary foods represents a critical transition in life, since it is crucial for optimal physiological development but may also represent a vector for pathogens invading the immature immune system of the developing child [7, 8].

The study of breastfeeding and weaning patterns through stable isotope analysis has become an increasingly important line of research in bioarchaeology [11–15]. Stable nitrogen and carbon isotopes have been used to interpret the duration of breastfeeding and weaning via cross-sectional analysis of archaeological populations, i.e. by taking bulk bone samples from children of different ages and comparing them with the adult values. In this way, changes in diet are inferred from sample trends, and differences between sites and socio-economic contexts are interpreted [10, 16]. Incremental studies of bone and dental samples provide higher-resolution dietary reconstructions [14, 17], and also reveal essential aspects of physiological stress and disease [18–21].

The study of infant diet and feeding practices has received little attention in the Baltics. Biomolecular analyses of weaning patterns were published for Swedish and Latvian prehistoric populations [22–24]. The health status of medieval and early modern Latvian communities (including children) has been addressed through paleopathology and stable isotope analysis [25–27]. Non-adult feeding practices have been marginally covered in wider dietary studies of Lithuanian medieval and early modern human groups [28, 29]. To date, no stable isotope study has analyzed infant diet and feeding practices in medieval and early modern Estonia.

The aim of this paper is to investigate non-adult diet in medieval and early modern Livonia, a historic region that mostly corresponds to present-day Estonia and Latvia, through carbon and nitrogen stable isotope analysis, with a special focus on early childhood breastfeeding and weaning practices. Bone samples from 176 individuals between the fetal and the 7–15 age ranges, recovered from four urban and rural cemeteries from Southern-Estonia (13^th^-17^th^ centuries AD), were analyzed to answer the following research questions:

1. What was the staple non-adult diet in this Southern-Estonian sample, and did it differ between urban and rural contexts?
2. Is a breastfeeding/weaning signal detectable in the dataset, and are there any differences in feeding practices between the different socio-economic contexts?

This is the first study focused on infant diet and feeding practices in the Eastern Baltics targeting different socio-economic contexts. It offers insights into childcare customs in a tumultuous period of Livonian history, and paves the way for future analyses of child health and nutrition in the area. Furthermore, this study provides a unique opportunity to explore the variability in feeding practices according to local cultural norms and socio-economic organization of daily lives.

### 1.1. Stable isotopes in the reconstruction of infant feeding practices

Carbon (δ^13^C) and nitrogen (δ^15^N) stable isotope analyses have been frequently used to study breastfeeding and weaning patterns in archeological samples. The basic principle is that a breastfeeding infant is consuming the mother’s tissues, showing a rise in δ^15^N values of about one trophic level (2–3‰) compared to the mother [11, 13]. This was best observed in studies of hair samples from modern populations [13, 30]. During the weaning process, the chemical composition of infant tissues is influenced by parameters resulting from exclusive breastfeeding, as well as from the consumed supplementary foods and drinking water. These parameters will change according to the variation of the proportional intake of breastmilk and complementary foods [4]. The gradual replacement of protein from breastmilk with protein from other foods, which are generally at a lower trophic level in respect to breastmilk, would result in infant δ^15^N values dropping to a level similar to those of the mother [11]. The same effect should be milder for δ^13^C values, with a trophic level shift of around 1‰ [13]. The infant is considered fully weaned when breastfeeding ceases. At this point, the chemical composition of tissues developing in a weaned child will depend primarily on his/her diet, drinking water, and metabolism. This may be similar to those of the mother and other adults in the population, or vary according to the access to different food sources affected by regional cultural practices and traditions [4].

Two ways of analyzing breastfeeding and weaning patterns are mainly performed in bioarchaeological studies. The cross-sectional analysis takes bulk bone samples from children of different ages, compares them between each other and with adult values, and obtains a broad picture of the weaning period in a population [2, 15, 31]. The incremental analysis is used to establish feeding practices at the individual level, with a life history approach. Here, non-remodeling tissues such as dentine are sampled in increments, revealing high resolution isotopic values for different formation times [14, 17–19, 32]. Recent application of incremental analysis has significantly contributed to the understanding of the effects of physiological stress, disease and malnutrition on isotopic values [20, 33], and has been backed up with modern population studies [34, 35].

The limitations of cross-sectional studies have been widely discussed. The main issues concern the equifinality of the isotopic interpretation, where particular values may be explained with several, equally probable scenarios [31, 36]. Other issues include the precision of the aging techniques, which may be over- or underestimating the effective age of the individuals especially in pathological cases, non-diet-related physiological processes causing isotopic shifts between mothers and offspring, and poorly understood bone turnover rates [6, 14, 19, 37]. Stable isotope values from one bone sample may have captured different dietary inputs, incorporating bone deposited in utero, during exclusive breastfeeding, and during weaning; the proportion of this bone with different compositions can vary throughout the skeleton according to different turnover rates. This often causes a remarkable delay in the recording of actual dietary changes [4, 7, 38]. Cross-sectional studies are also greatly affected by the number and age distribution of non-adults in the cemetery sample, often resulting in unbalanced datasets and lack of key age ranges [4]. Similar studies request large numbers of individuals and can be therefore performed only on larger cemetery samples with relatively good preservation conditions. Individuals not preserved enough to allow age estimation will be excluded from the analysis, causing further loss of data [4]. Furthermore, estimating the weaning time inevitably assumes that the females in the population are representative of the mothers, and an overall homogeneity of the adult female diet. It is therefore common to infer a single nursing pattern for an entire sample or for particular groups of individuals within the dataset [4, 39]. This cuts out individual variability and cultural practices dictated by social status, gender roles, or health conditions [16]. As specific infant–mother couples are rarely identified, the overall non-adult values are compared to an overly homogeneous adult baseline with the risk of misinterpreting those outliers with particularly high or low values [4]. As a result of the osteological paradox, the children in the cemetery are non-survivors that may have not experienced the same feeding practices as the survivors, or whose deaths may have been caused by different weaning trajectories [14, 18, 19, 40]. Similar issues can be limited by adopting incremental methods, which record life stages other than those immediately before death. Thus, different high-resolution snapshots of diet and physiological stress can be obtained through strategic sampling [18–20].

However, despite the limitations mentioned above, the cross-sectional approach remains the most useful and cost-effective way of creating general models of population weaning strategies. In combination with incremental techniques, it has the potential to provide the most nuanced set of data for studies of past feeding practices [31].

### 1.2. Historical background: Food consumption and child feeding practices in Livonia

**Fig 1:**
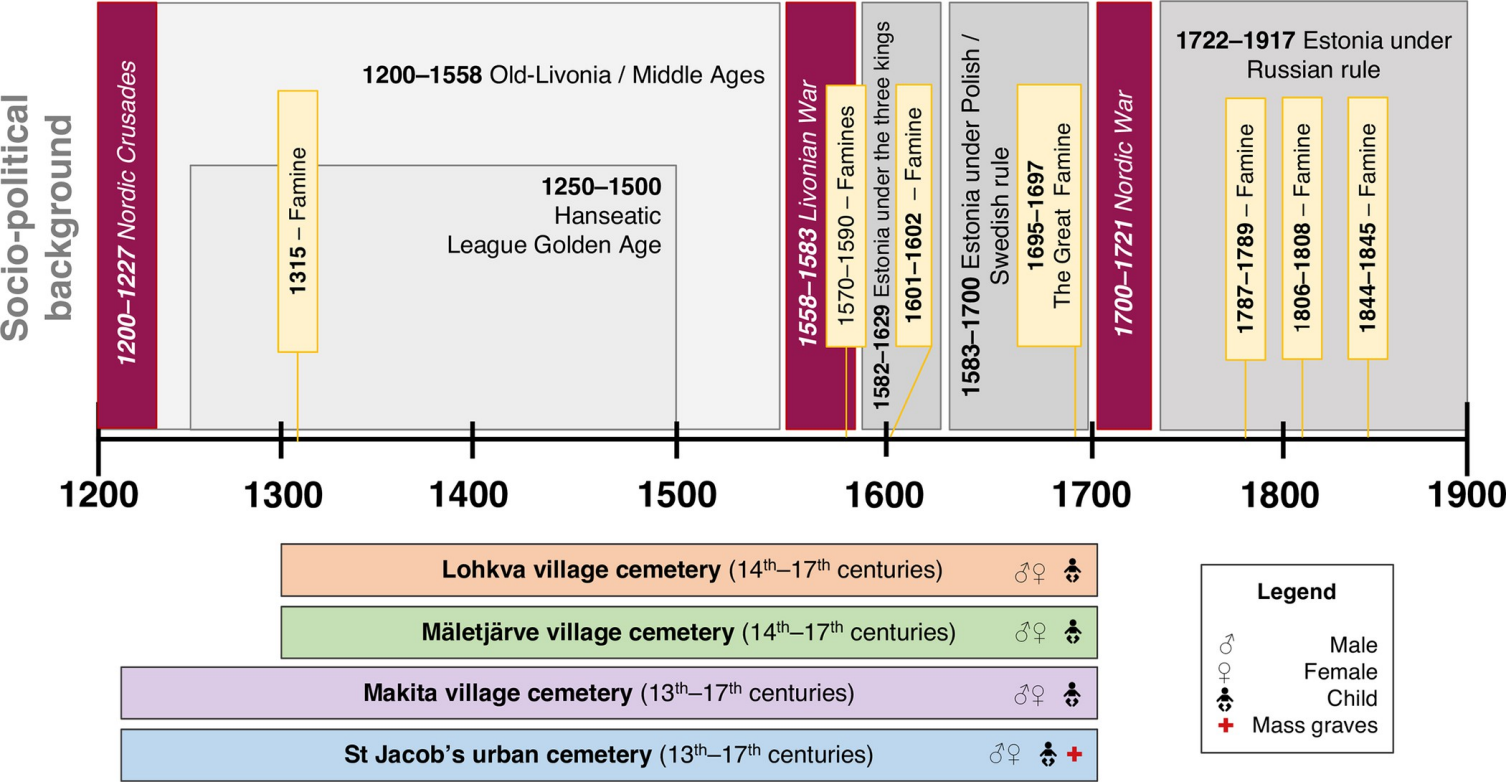
Historical context of this study. Major socio-political events and the corresponding timeframes of burials in the analyzed cemeteries.

Southern-Estonia formed a central part of medieval (13^th^–16^th^ centuries AD) and early modern (16^th^–18^th^ centuries AD) Livonia. Historical records testify to a series of socio-cultural processes such as foreign conquests and supplanting of local politico-economic powers by several non-local stakeholder groups (Germans, Poles, Swedes), and development of urban settlements with typically “European” features (Fig 1) [41]. A major disunity between the foreign upper class (the *Deutsch*, *i*.*e*. Germans, who were traders, clergy, and honorable craftsmen) and the local lower class (the *Undeutsch*, *i*.*e* non-Germans or Estonians, including craftsmen considered less honorable and poor citizens, mostly from the hinterlands) has been reported among the urban populations [41–43]. Furthermore, several subsequent famines and epidemics, often intertwined with warfare, caused large population losses [41, 44] (Fig 1). It can be assumed that the upheavals of this long and tumultuous period had a considerable impact on the socio-economic conditions of the local populations, among which dietary practices and food availability must have played a crucial role in everyday lives of adults and children.

We can take a glance into the general dietary habits of medieval and early modern Estonian populations through a plethora of archaeological and historical sources [45–49]. The Hanseatic trade allowed importation of several foreign products into Livonia, resulting in a wider food variety in the city as compared to the village. The wealthy German upper class enjoyed higher quality resources and exotic imported foods, such as spices (salt, pepper, ginger, saffron, cinnamon, nutmeg, and sugar), herbs, cheese, game, wine, almonds, and imported fruits (raisins, dates). However, it appears that dietary staples (bread, meat, fish) were quite similar for all social groups, with the lower classes integrating them with more vegetables (onion, bean, pea, carrot, cabbage, turnip), butter, and fermented dairy products [49, 50].

The medieval city dweller typically obtained the necessary food from the household, since the preparation of bread, mead, beer, and meat/fish was mainly carried out at home [50]. Small garden plots, as well as pigs and poultry, were kept inside and near the town walls, while livestock was raised in grasslands outside the city boundaries. Fish was common in both upper and lower classes and frequently consumed during fasting, with herring, salmon, and Atlantic cod being the main imported fish in urban and rural areas; freshwater fish (bream, pike, perch, carp) were also bred in town moats [45, 47, 48, 51, 52]. A smaller range of foods was available in rural areas, which mostly imported salt, herrings, and hops; food preparation techniques also varied significantly between towns and countryside [47].

Unfortunately, few historical studies regarding childhood in medieval and early modern Livonia exist, and no specific information concerning the duration of breastfeeding or weaning is available [50, 53]. This severely limits our knowledge of feeding customs and childcare in the area.

## 2. Materials and methods

### 2.1. The urban cemetery of St Jacob in Tartu

**Fig 2:**
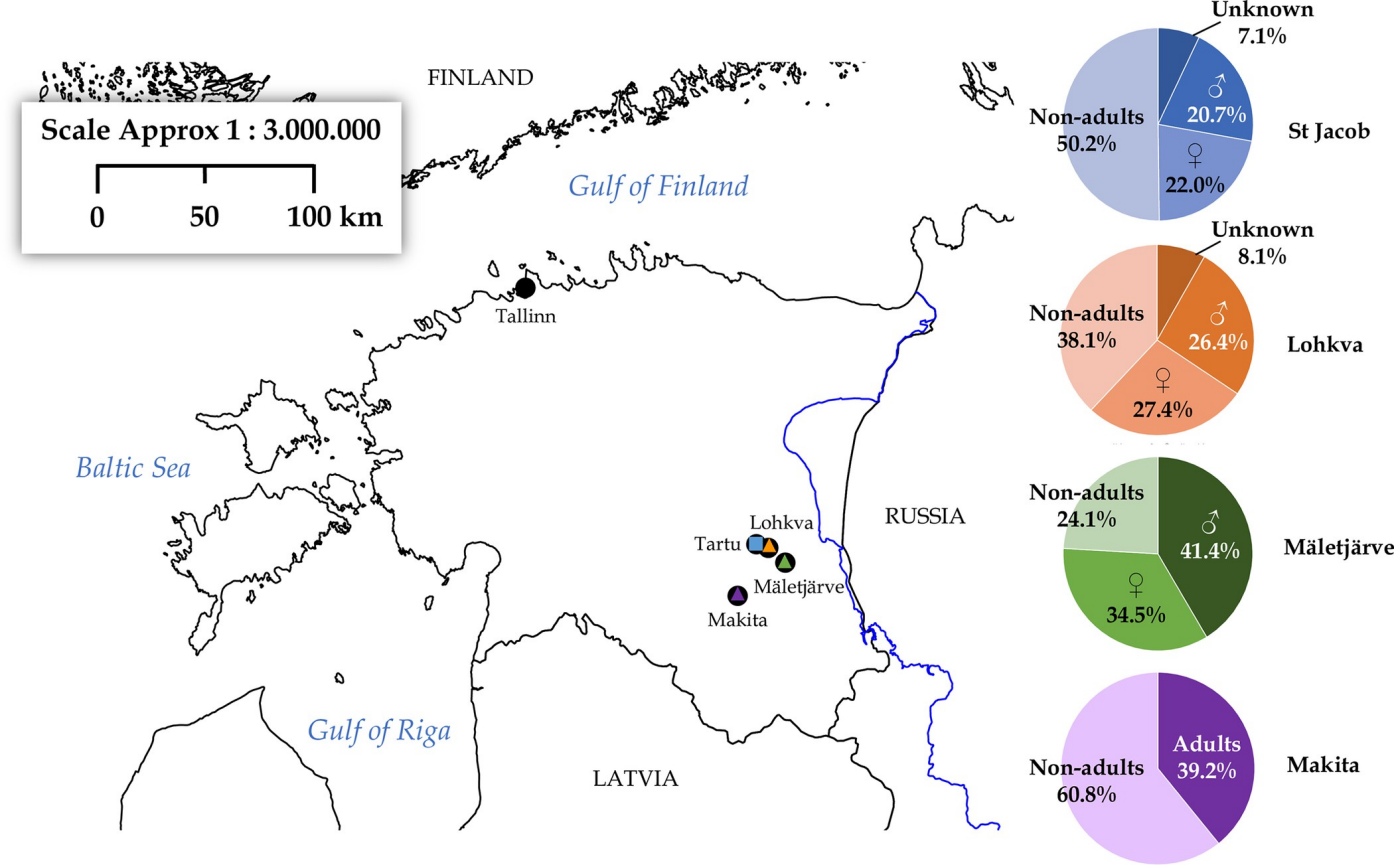
Location of the studied rural and urban cemeteries. Squares indicate urban contexts, while triangles represent rural areas. The demographic analysis for each site is reported in the pie charts, which show a high variability in the percentage of non-adult individuals in each context [54, 57, 59, 60]; (Malve, original data). ♂ = adult males; ♀ = adult females; non-adults = individuals under 17 years of age; Unknown = adult individuals for whom sex estimation was not possible.

St Jacob’s cemetery is one of the medieval and early modern suburban cemeteries discovered in the city of Tartu. This was the third largest town in Livonia, with a population of 5,000–6,000 people in the mid–16^th^ century [54]. The churchyard was located outside the city walls, and it is assumed that inhabitants of Tartu’s medieval suburbs, part of the lower status urban population, and migrants from close rural areas were buried there [55]. Archaeological excavations (2010–2017) revealed at least 614 individuals. Based on the associated artifacts, the cemetery was dated to the mid-13th–late 16^th^/early 17^th^ centuries. The burials were mostly simple graves with no coffin or grave goods, and the dead were buried with their heads turned westwards. The proportion of non-adults in St Jacob’s cemetery (~50%, Fig 2) is consistent with other coeval Estonian churchyards [56–58]. The bioarchaeological analysis of a non-typical mass burial of babies discovered outside St Jacob’s cemetery walls has already been reported by the first author [59].

### 2.2. The rural cemeteries

Three village cemeteries were selected from the rural surroundings of Tartu: Lohkva, Mäletjärve, and Makita. The medieval/early modern settlement of Lohkva (11^th^–17^th^ century) is located southeast of Tartu. Rescue excavations were carried out in 2011, unearthing the ancient settlement and the village cemetery [60]. Ninety-nine burials were recovered, most of which were single inhumations in a supine stretched position, with the head turned westwards; most burials included coffin wood and/or nails [60]. The grave goods consisted mainly of coins, rings, brooches, and knives dated to the second half of the 16^th^ and to the 17^th^ century. However, one burial contained a 14^th^–16^th^ century needle-sheath, suggesting that the cemetery was already in use in the medieval period [60]. The rather low proportion of non-adults (38.1%) was considered exceptional in the context of Estonian medieval/early modern cemeteries [56, 57, 61]. Of interest also was the high number (51%) of mature and elderly adults (35+ years), suggestive of relative longevity in this community.

The village cemetery of Mäletjärve, excavated in 1984, is located southeast of Tartu. It was in use between the 14^th^ and the 17^th^ centuries, based on the recovered artifacts (coins, rings, bronze jewelry), but most graves are from the 16^th^ century [62]. Due to the rescue nature of the excavation, a limited number of skeletons (29, all supine with head turned westwards) was available for osteological investigation. Out of these, the non-adult sample formed 24.1% (Fig 2).

The rural cemetery of Makita is located in the Tartu district near Otepää, and was excavated in 1986–1987. The numerous artifacts (including coins, brooches, jewelry, and ornaments) date the cemetery between the 13^th^ and the 17^th^ centuries [63]. The burials were mainly single inhumations in a supine stretched position with the head turned west, and the hands in variable positions. Many burials included coffin wood and/or nails and were often delimited by stones inside and on the grave margins [63]. A hundred and twenty-five skeletons were recovered and divided in two chronological groups based on artifacts belonging to the 13^th^–15^th^ and the 15^th^–17^th^ centuries AD. Non-adults constituted 60.8% of the recovered burials (Fig 2). The demographic composition is comparable to that of other coeval Estonian cemeteries [57, 63].

### 2.3. Bioarchaeological methods and sample selection

Ethical approval was granted by the Department of Archaeology, Institute of History and Archaeology, University of Tartu (UT). According to the research procedures in place at the institute, no permits were required for the described study, which complied with all relevant regulations. The specimens studied are stored among the UT Institute of History and Archaeology archaeological collections, Tartu, Estonia. A detailed anthropological study was carried out on the non-adult individuals. Age at death was estimated using standards based on tooth formation and eruption [64–67], bone fusion [68, 69], and long bone diaphyseal length [70–73]. In accordance with Lewis [74], the youngest individuals were included in the following age categories: *fetus* (under 28 gestation weeks), *perinate I* (preterm, 28–37 gestation weeks), *perinate II* (full term, 37–42 gestation weeks), and *neonate* (from 42 weeks to around a month old). The term “perinate” defines the individuals who died around the time of birth, with the *perinate II* category including possible full term and post-term births. Those aged younger than 37 weeks (*perinate I*) were possibly either aborted (death in utero), stillborn (death after 28 weeks of gestation), or dead immediately after birth, in the case of pre-terms or babies that were small for gestational age [75].

A hundred and seventy-six non-adults were sampled from the urban and rural contexts (Table 1). Exceptionally preserved ribs or rib fragments were preferred for sampling. However, when these were unavailable/pathological, long bones (mainly upper limbs) or other skeletal elements were chosen. From these, 60–100 mg of bone pieces and powder were collected with a Saeyang Marathon-N7 drill using new precleaned drill bits for each sample at the UT Department of Archaeology laboratory.

**Table 1:** Assemblage of this study.

Site/Dates	Fetus	Perinate I	Perinate II	Neonate	Infant 0–1	Child 1–7	Child 7–15	Selected samples for SIA	Samples meeting quality criteria
**St Jacob (13**^**th**^**-17**^**th**^ **century)**	5	25	28	2	3	12	7	82	68
**Lohkva (14**^**th**^**-17**^**th**^ **century)**	0	1	2	1	1	10	5	20	19
**Mäletjärve (14**^**th**^**-17**^**th**^ **century)**	0	0	0	0	0	3	4	7	2
**Makita (13**^**th**^**-17**^**th**^ **century)**	1	2	1	3	7	37	16	67	51
**Total**	6	28	31	6	11	62	32	176	140

Non-adult skeletal assemblage sampled for SIA and number of individuals with good collagen quality for each age category.

The principal limitation of this dataset is represented by the age-at-death composition of the urban versus the rural samples. The funerary record of the urban sample is skewed towards the youngest individuals, while the rural samples tend to represent older children, lowering the informative power of the statistical comparisons between sites. This is an unfortunate consequence of performing cross-sectional studies on archaeological populations, which mostly make a biased proxy of the original human communities [4]. Intrinsic factors (such as different biological aspects of the human bone structure, and physiological processes), and extrinsic factors (taphonomic effects of the burial environment such as chemical composition of the underground/rain waters, soil pH and texture, oxygen levels, flora, fauna, excavation history, and post-excavation curation) affect sample representativity, especially with regard to non-adult individuals [76–79]. In addition, cultural factors such as funerary customs and differential burial position, grave depth, or body treatment may cause bias in the available skeletal population [76–79].

Regarding our dataset, the large number of perinates from St Jacob’s cemetery come from mass graves outside of the cemetery walls specifically dedicated to perinates, indicating the choice of a specific burial area for particular population groups [59]. The sample is a unique finding in Estonian urban contexts from the archaeological and paleopathological points of view, and little comparative material has been found. The discovery of this “children’s corner” influences the overall representation of perinates in this site, but creates a unique opportunity to analyze this vulnerable social group. Other external biases could have resulted from the dataset at hand. For instance, as a result of the excavation conditions, the rescue works at Mäletjärve cemetery (sampled in its entirety) led to the recovery of only 29 individuals, among which non-adults were less represented (the badly preserved were also left on-site) and babies were absent. This may also be indicative of bias in the initial cemetery population, since it is likely that babies were either buried elsewhere, or the area dedicated to children was not excavated. The other rural cemeteries also presented lower numbers of babies and infants compared to the urban area. Yet, acknowledging these sample biases, the limited representation of the youngest age ranges in the overall archaeological record further highlights the unique and informative value of St Jacob’s perinates. The successive collagen extraction also contributed to the loss of information from rural babies and infants, since the samples that did not meet the quality criteria for collagen preservation were excluded from the analysis. However, despite this inevitable imbalance in the age composition of the dataset, the differences between rural and urban individuals are still appreciable, and the sample is large enough to retain statistical power.

### 2.4. Collagen extraction and stable isotope analysis

The samples were processed at the Archemy Laboratory, UT. Collagen extraction was performed using the modified Longin method [80]. Bone samples were demineralized in 0.25M hydrochloric acid solution (HCl) at room temperature for 48 hours. The demineralized samples were rinsed with deionized water, and collagen was gelatinized in the oven at 58°C for 16 hours in tubes with 0.01M HCl. The resulting solution was filtered with Whatman® nitrocellulose membrane filters (pore size 5 μm) to remove any insoluble residue, and freeze-dried.

Stable carbon (δ^13^C) and nitrogen (δ^15^N) isotopic compositions were measured using an automated carbon and nitrogen elemental analyzer isotope ratio mass spectrometer (EA-IRMS) in the Department of Geology, UT. Bone collagen samples were weighed into tin capsules (~1.0–1.2 mg) and combusted in a Thermo Flash^TM^ HT EA with the introduction of separated N2 and CO2 to a Delta V plus via a ConFlo IV interface. The analysis was run in duplicates (i.e., two samples of collagen from the same individual), and the final values averaged. The data were calibrated against international standards from IAEA (for nitrogen IAEA N-1, δ^15^N_AIR_ = +0.4‰, IAEA N-2, δ^15^N_AIR_ = +20.3‰, USGS25, δ^15^N_AIR_ = −30.4‰, and for carbon IAEA CH3, δ^13^C_VPDB_ = −24.72‰, and IAEA CH6, δ^13^C_VPDB_ = −10.449‰). The results are expressed using the delta notation in parts per thousand (per mil or ‰) [81] relative to the international marine limestone VPDB standard for carbon and AIR for nitrogen. The long-term stability error for the isotope ratio measurements, estimated from repeated measurements of international and laboratory standards, was better than ±0.2 ‰ (1sd) for nitrogen, and ±0.1 ‰ (1sd) for carbon.

As references, the mean values of the adult population from these cemeteries are used. The adult individuals (n = 25) were sampled and analyzed by the first author. The local and temporally coeval faunal stable isotope results were provided by Aguraiuja-Lätti (original data), and Malve (original data).

### 2.5. Statistical analysis

Statistical analysis was performed using PAST 4.09 software [82]. A Shapiro-Wilk test of normality was initially used before selecting the appropriate comparative tests (S1-S4 Tables in S1 File). The urban counterpart of this study has shown a normal distribution. Although the rural part is not normally distributed, the single rural subgroups varied due to limited sample size. Hence, a nonparametric Mann–Whitney *U* test and a Kruskall–Wallis test were used for comparison of two or more than two samples (ex. among cemeteries/age ranges). Possible statistical outliers were identified using the IQR (Tukey’s Interquartile Range) criterion.

## 3. Results

### 3.1. Collagen quality

Out of all 176 samples, 140 (79.55%) presented sufficient collagen preservation according to standard quality control methods: C:N atomic ratio between 3.07 and 3.59 (mean 3.31 ± 0.09); weight percent collagen yield ≥1%; weight percent carbon (%C) >13%; weight percent nitrogen (%N) >4% [83–87]. The mean carbon (32.23 ± 9.99 wt% C, 1sd) and nitrogen (11.42 ± 3.63 wt% N, 1sd) contents indicate good collagen preservation. Thirty-three individuals from all contexts did not provide enough collagen for analysis, and only three with sufficient collagen did not meet the quality control criteria (raw data in S5 Table in S1 Table).

### 3.2. Non-adult δ^13^C and δ^15^N values for the whole sample and by context

The δ^13^C values of the whole sample of 140 non-adults range from -22.42‰ to -19.23‰ (mean ± sd = -21.10 ± 0.50‰), while the δ^15^N values range from 7.72‰ to 16.05‰ (mean ± sd = 11.79 ± 1.72‰).

**Fig 3:**
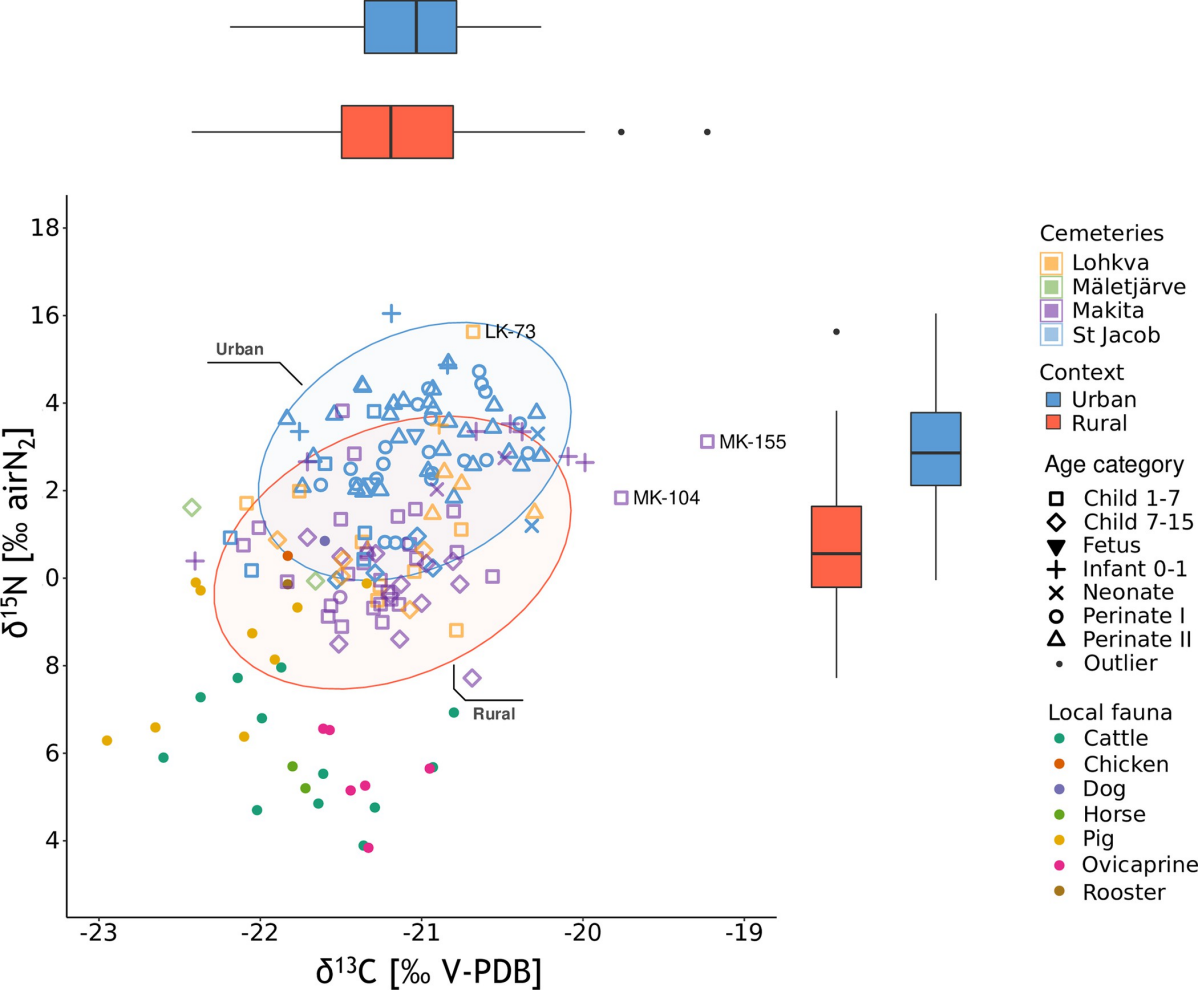
Carbon and nitrogen stable isotope values of the samples colored by the cemeteries used in this study. The urban and rural distributions are separated in the boxplots. The values of local fauna are added as references (Aguraiuja-Lätti, original data; Malve, original data). The ellipses are drawn with 95% confidence intervals, and the outliers related to the urban and rural samples were determined using the IQR criterion.

**Fig 4:**
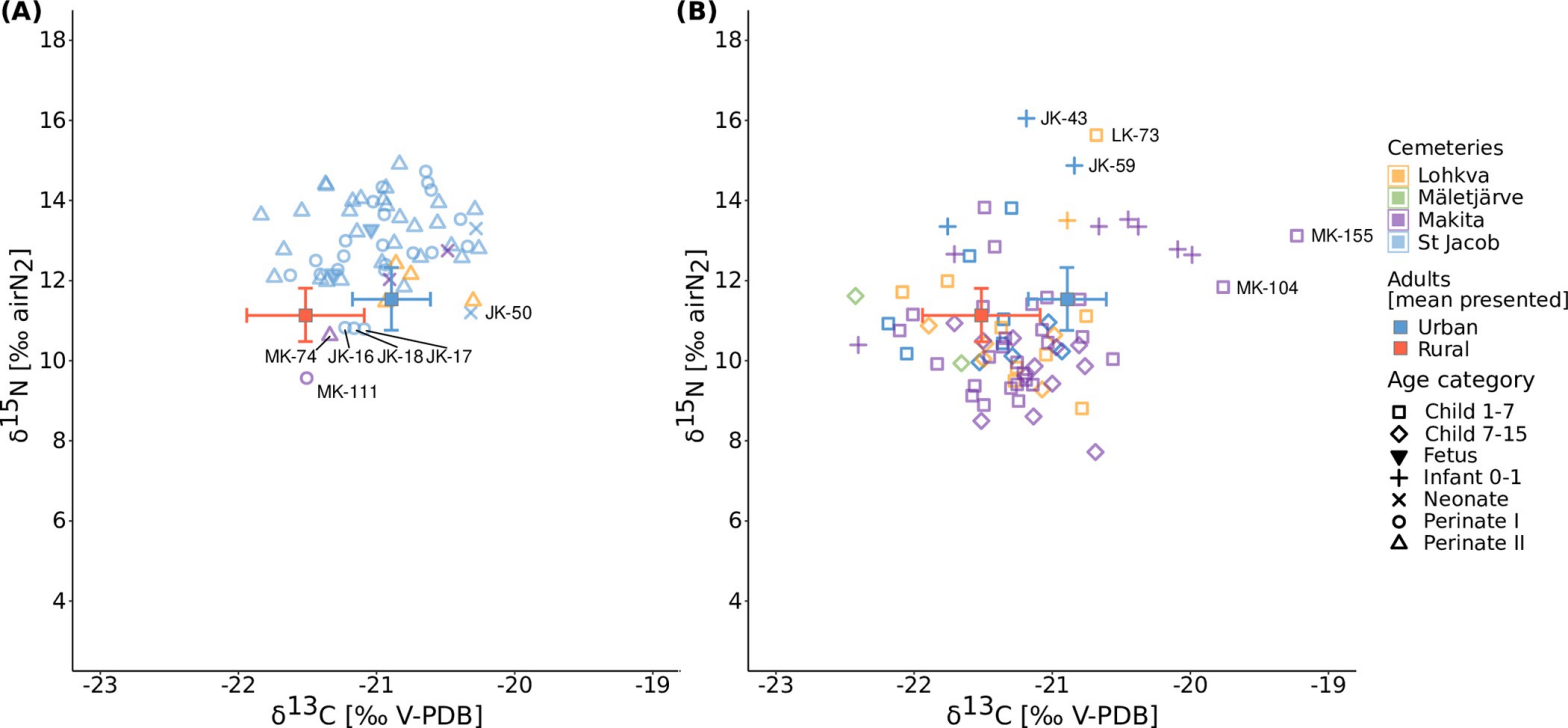
Carbon and nitrogen stable isotope values of the samples colored corresponding to the cemeteries used in this study, plotted with the urban and rural mean adult values (including both males and females). (A) Scatter plot of the fetuses, perinates I, perinates II, and neonates. (B) Scatter plot of the infants and older children. The ID codes of the outliers and individuals with anomalous values discussed in the text are included.

The δ^13^C values of the urban non-adults range from -22.19‰ to -20.26‰ (mean ± sd = -21.06 ± 0.43‰) and the δ^15^N values range from 9.96‰ to 16.05‰ (mean ± sd = 12.82 ± 1.32‰). For the rural non-adults, the δ^13^C results range from -22.42‰ to -19.23‰ (mean ± sd = -21.15 ± 0.56‰) and the δ^15^N values range from 7.72‰ to 15.63‰ (mean ± sd = 10.82 ± 1.48‰). Comparing the contexts, the nitrogen values show a notable variability reflecting the differences in the age range distribution between sites, as well as the presence of outliers (Table 2, Figs 3 and 4).

**Table 2:** Descriptive statistics of urban and rural non-adults for each age range.

		St Jacob’s urban cemetery							
	N samples	Mean δ^13^C ‰	Min	Max	sd	Mean δ^15^N ‰	Min	Max	sd
**Fetus**	2	-21.18	-21.31	-21.04	0.14	12.72	12.17	13.27	0.55
**Perinate I**	22	-20.97	-21.62	-20.34	0.34	12.81	10.79	14.73	1.11
**Perinate II**	29	-21.01	-21.84	-20.26	0.42	13.28	11.84	14.91	0.84
**Neonate**	2	-20.30	-20.32	-20.28	0.02	12.25	11.20	13.30	1.05
**Infant 0–1**	3	-21.26	-21.76	-20.84	0.38	14.76	13.35	16.05	1.10
**Child 1–7**	6	-21.64	-22.19	-21.29	0.35	11.50	10.18	13.81	1.29
**Child 7–15**	4	-21.19	-21.53	-20.93	0.23	10.32	9.96	10.96	0.38
**Total**	68	-21.06	-22.19	-20.26	0.43	12.82	9.96	16.05	1.32
		**Rural cemeteries**							
**Fetus**	0	0	0	0	0	0	0	0	0
**Perinate I**	1	-21.51	-21.51	-21.51	0	9.57	9.57	9.57	0
**Perinate II**	5	-20.84	-21.34	-20.30	0.33	11.63	10.63	12.42	0.63
**Neonate**	2	-20.70	-20.91	-20.49	0.21	12.39	12.03	12.74	0.36
**Infant 0–1**	8	-20.82	-22.40	-19.99	0.78	12.77	10.39	13.52	0.97
**Child 1–7**	37	-21.19	-22.10	-19.23	0.55	10.69	8.81	15.63	1.45
**Child 7–15**	19	-21.30	-22.42	-20.69	0.42	9.95	7.72	11.61	0.92
**Total**	72	-21.15	-22.42	-19.23	0.56	10.82	7.72	15.63	1.48

Max = maximum; Min = minimum; N = number; sd = standard deviation.

The isotopic values for the urban sample were higher compared to the rural sample by 0.09‰ for δ^13^C, and by 1.99‰ for δ^15^N (Fig 3). Since the δ^15^N values of the rural children do not have a normal distribution (S1 and S3 Tables in S1 File), a nonparametric Mann–Whitney test was performed to compare them. No significant difference was observed between the urban and rural samples in the δ^13^C values (U = 2118, z-score = 1.374, p-value = 1.170), but a significant difference was observed for the δ^15^N values (U = 778, z-score = 6.961, p-value = 3.388^e-12^).

A Kruskall–Wallis test was performed to compare the δ^15^N and δ^13^C values between children from different sites. No significant difference between sample medians was observed for δ^13^C. The Mann–Whitney tests between group pairs showed a significant difference between Mäletjärve and the other sites (S6 Table in S1 File), with the former being lower in respect to St Jacob (0.98‰), Lohkva (0.90‰), and Makita (0.93‰).

For δ^15^N, a highly significant difference was observed between sites. The Mann–Whitney tests between group pairs highlighted a significant difference between St Jacob’s and the other cemeteries (S7 Table in S1 File). The former shows much higher δ^15^N in respect to Lohkva (1.67‰), Mäletjärve (2.04‰), and Makita (2.11‰), confirming the results of the Mann-Whitney test on the urban and rural whole samples.

### 3.3. Non-adult δ^15^N values compared to the adult female baseline

The δ^13^C values of the urban women range from -21.47‰ to -20.48‰ (mean ± sd = -20.02 ± 0.29‰) and the δ^15^N values range from 10.01‰ to 12.67‰ (mean ± sd = 11.18 ± 0.80‰). The δ^13^C values of the rural women range from -22.39‰ to -20.63‰ (mean ± sd = -21.66 ± 0.45‰) and the δ^15^N values range from 10.39‰ to 12.31‰ (mean ± sd = 11.17 ± 0.63‰) (see summary statistics for women in S8 Table in S1 File). It must be pointed out that the number of female values (20−35 and 35−50 age categories) used as a comparative baseline for children is relatively small (n = 8), due to the sample availability and collagen preservation. However, we considered it suitable for a preliminary comparative baseline.

According to the existing literature, it is assumed that individuals being breastfed would show isotope values higher than the average adult females of about 1‰ in δ^13^C and 2–3‰ in δ^15^Ν [13]. Most of the non-adults in this study show elevated δ^15^Ν values compared with the general adult means, with younger individuals homogeneously displaying higher values (Fig 4A), and older children scattering around the adult means (Fig 4B).

**Fig 5:**
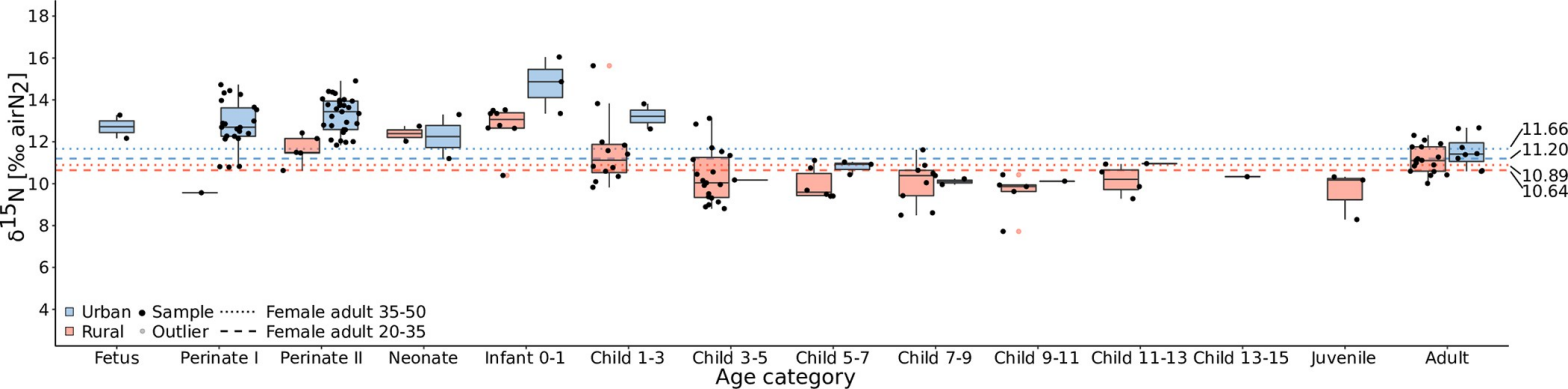
Boxplot of the δ^15^N values of rural and urban children. The blue color indicates urban individuals, while the pink color indicates rural individuals. The non-adult values were plotted against the mean δ^15^N values of the urban and rural female individuals at the age of 20–35 and 35–50 years, indicated in the graph with the blue and pink dotted and dashed lines. The children in the 1–7 and 7–15 categories were further divided into smaller 2-years groups to provide higher resolution to the data. Statistical outliers have been determined for each age group according to the IQR criterion.

Compared to the adult females, the fetal and preterm age categories show δ^15^Ν values between 1 and 2‰ higher, the full-term babies tend to exceed the 2‰ threshold, and the infants (0–1 years) reach the expected ca. 2–3‰ increase indicative of a breastfeeding signal, touching the highest values of ca. 3.7‰ when urban infants and women are compared (Table 3). A somewhat unexpected result concerns neonates, where the mean values are closer to the perinates I (ca. 1–2‰) than to the infants. However, neonates are highly underrepresented in this sample (n = 4), and their ratios will be discussed separately. The children in the 1–3 age categories show δ^15^Ν values enriched by ca. 2‰ with respect to the females, dropping to values within 1sd from the female mean after the age of 3. Most of the older children show δ^15^Ν values lower than the women’s means (Fig 5).

**Table 3:** Summary statistics of non-adult isotopic values for each subcategory.

	St Jacob’s urban cemetery			Rural cemeteries		
	N samples	Mean δ^13^C ‰	Mean δ^15^N ‰	N samples	Mean δ^13^C ‰	Mean δ^15^N ‰
**Females 20–35**	2	-20.76	11.09	2	-21.15	10.49
**Females 35–50**	3	-21.11	11.05	1	-21.80	11.76
**Fetus**	2	-21.18	12.72	0	0	0
**Perinate I**	22	-20.97	12.81	1	-21.51	9.57
**Perinate II**	29	-21.01	13.28	5	-20.84	11.63
**Neonate**	2	-20.30	12.25	2	-20.70	12.39
**Infant 0–1**	3	-21.26	**14.76**	8	-20.82	**12.77**
**1–3**	2	-21.45	13.21	12	-21.10	11.56
**3–5**	1	-22.05	10.18	19	-21.22	10.36
**5–7**	3	-21.63	10.80	6	-21.29	9.98
**7–9**	2	-21.23	10.10	9	-21.42	10.06
**9–11**	1	-21.29	10.12	5	-21.16	9.51
**11–13**	1	-21.03	10.96	4	-21.30	10.16
**13–15**	0	0	0	1	-20.97	10.33
**Juvenile 15–20**	0	0	0	3	-21.47	9.59

To better observe the possible differences, the children in the 1–7 and 7–15 categories were further divided into smaller 2-years groups, and their means (pink color) were compared to those of the women in the 20–35 and 35–50 age categories (highlighted in purple). The standard deviations are reported in the supporting information.

## 4. Discussion

### 4.1. Staple non-adult diet and differences between urban and rural contexts

The mean δ^13^C values suggest that the staple non-adult diet in medieval and early modern Southern Estonia was based on a terrestrial C_3_ ecosystem, dominated by C_3_ plants or C_3_ plant consumers and their products. These results are in line with δ^13^C data from other coeval Baltic sites [43, 88, 89], and reflect the common subsistence in that period, with cultivations of barley, oats, rye, legumes, vegetables, fruits and berries constituting the staple [47, 49]. This can be also inferred by comparing the population values with the animal baseline, which reasonably reflects the food chain in the area (Fig 3; S1 Fig in S1 File).

No significant difference in non-adult carbon values was seen between urban and rural areas, with the former being slightly higher than the latter (0.09‰). This suggests an overall similar diet for children living in different contexts. The small difference in δ^13^C may be attributable to slight variations in food habits and subsistence, confirming previous studies reporting on a staple diet including mainly C_3_ foodstuffs in both town and countryside contexts [43, 49]. From this baseline, some townspeople showed greater isotopic variation, possibly reflecting social and ethnic differences, and/or individual dietary choices. For instance, they may have had access to small amounts of C_4_ foods, such as millet [43]. Although millet was not particularly common in Estonia, it is assumed that it was consumed by urban middle classes and allegedly imported to Tartu from the Southern Baltics [46]. Although no written source mentions any dietary difference between towns and countryside, different trends in food routes and the influence of the local feudal lord likely affected subsistence strategies in urban areas. This may explain the slight variation in δ^13^C between Mäletjärve and the other rural sites. The lower values in Mäletjärve may be due to lower amounts of available C_4_ plants, and/or to fewer animal products consumed by these children. It must also be pointed out that only two individuals in the 7–15 range provided ideally preserved samples for analysis, and this undermines the interpretation. However, this trend is also shown by the adult population (S1 Fig in S1 File), so it likely reflects a general population pattern or secular changes between sites, rather than a specific diet in Mäletjärve.

The δ^13^C values in non-adults (mean ± sd = -21.10 ± 0.50‰) are comparable with the adult values (mean ± sd = -21.31 ± 0.47‰), especially with regard to those in the 1–7 and 7–15 age categories (Fig 4). Hence, it can be assumed that they received proportions of C_3_ foods similar to adults once completely weaned. This interpretation appears to be consistent with historical sources (mainly normative documents) from which it is inferable that children were put to work quite early, already at 5–6 years of age, and given simpler tasks in the household. For instance, one of the most common tasks of child laborers was herding. The common principle was that hard workers could not be under-nourished and required more food, so it is reasonable to assume similar diets for children and adult workers. Yet, it was also stated that children were usually given less meat [53]. This may explain the lower δ^15^N values of older children and adolescents (Fig 4B).

The δ^15^N values show the greatest variability and spread in both contexts. The δ^15^N values in non-adults (mean ± sd = 11.79 ± 1.72‰) are similar to the adult values (δ^15^N mean ± sd = 11.26 ± 0.69‰), although they show greater variability due to the wide age range selection. As can be inferred from the comparison with the animal baseline, these results imply that adults and children in South-Estonia likely had wide access to animal products, possibly relying on small amounts of higher trophic level proteins, such as fish. In particular, human individuals show δ^15^N values elevated by 4.94‰ (δ^13^C = 0.28‰) compared to cattle, by 5.81‰ (δ^13^C = 0.45‰) compared to horses, by 5.33‰ (δ^13^C = 0.00‰) related to ovicaprines, and between 0.41 and 2.93‰ (δ^13^C between 0.28 and 0.86‰) in respect to omnivores, with the dog showing the greatest similarities to humans (δ^13^C = -21.60‰, δ^15^N = 10.85‰). These values, together with the similarity with omnivores, therefore seem to show a trophic effect that is compatible with consumption of protein sources not only from plants, but also of animal origin. This would also be in line with historical sources reporting a consistent presence of animal products in the diet of Estonians, including fish [48–50, 90]. Marine and freshwater fish were commonly consumed in Estonia, and since their δ^15^N values are generally above 10‰, fish consumption may explain higher human δ^15^N values [20, 90]. Similar scenarios were also inferred by previous studies in the Baltics: although Swedish and Finnish sites report slightly higher mean values (closer to 13‰), interpreted as higher marine fish consumption [89, 91, 92]; other Baltic sites displayed values comparable to this study, interpreted as the result of freshwater fish consumption and negligible marine input [49, 88, 90]. However, due to the low salinity of the Baltic sea, marine fish here show lower C and N isotopic values, so it is complicated to discern between a diet with low amounts of imported marine fish, and one with higher amounts of local marine and freshwater fish [51, 89, 90]. It is known that the consumption from freshwater sources in Tartu was consistent in medieval/early modern periods, since the city is located on the Emajõgi River and is close to Lake Peipsi [47, 48]. Furthermore, a previous study reported that marine resource consumption did not differ significantly between Tallinn and Tartu, as this was the main Hanseatic trading center in Southern Estonia [49].

The reliance on manured plants (or animals fed on them) may be undetectable from isotopic values. Using animal values as proxies, the herbivore nitrogen values tend to be quite low (between 4–6‰), while the increased human (and omnivore) values are compatible with a standard trophic effect [93–96], with an increase of nitrogen values (here around 5‰) in respect to herbivores, and similarities with omnivore animal values (S1 Fig in S1 File). Hence, it seems that either these populations did not heavily rely on manuring, the animals were not feeding on manured plants but were rather freely grazing, or the degree of manuring was not high enough to influence bone δ^15^N values [49].

Besides standard dietary variations, it must also be considered that this is an exclusively non-adult sample. A statistically significant difference between urban/rural areas was observed for δ^15^N values (1.99‰ higher in the urban sample), a general trend detectable in both babies (Fig 4A) and older children (Fig 4B). Several factors may explain this difference. First, the sample from St Jacob displays the highest number of perinates, which normally have higher δ^15^N values compared to adult means. Different δ^15^Ν in perinatal bone collagen are likely to reflect in-utero values, and the positive nitrogen balance caused by pregnancy and rapid fetal development can result in a possible δ^15^N increase. However, it is unclear how physiological variations in protein metabolism between mother and fetus influence δ^15^N values in fetal bone collagen [10, 13, 34, 97]. On the other hand, high δ^15^N values have also been correlated to physiological stress and disease [18, 98–100]. Most of the perinates from St Jacob’s were recovered from atypical mass graves located outside the cemetery boundary. Many of these babies displayed skeletal lesions attributable to systemic metabolic disease, interpreted as resulting from numerous epidemics and famines affecting pregnant and nursing mothers [59]. Hence, the effects of diseases such as congenital scurvy and/or rickets transmitted by undernourished mothers on δ^15^N values cannot be ruled out, especially with regard to those babies showing δ^15^N over 13‰. Nonetheless, although the rural perinates are much fewer than those from Tartu, their values tend to reflect the general urban/rural trend of the whole non-adult sample, with lower δ^15^N compared to rural babies (Fig 4A). Therefore, in addition to mother/fetus physiological variations and perinatal stress, possible dietary differences among pregnant and lactating mothers are still a possible explanation.

Second, if the perinatal sample is excluded, the non-adult δ^15^N values in St Jacob’s still tend to be higher compared to the rural sites (Fig 4B). This strengthens the assumption that there were effective dietary variations between town and countryside dwellers. The former possibly had access to a wider range of foodstuffs, including imported marine products, given the importance of Tartu in the Livonian trading system [47]. This can be confirmed by comparative data from other Baltic sites, in which villagers also display lower isotopic values compared to urban communities; similar patterns were interpreted as indicative of a terrestrial diet with minor aquatic components [28, 29, 49].

It is unlikely that the rural population consumed fewer animal products than the lower status people in urban Tartu. According to Aguraiuja-Lätti & Lõugas [49], urban livestock was probably fed differently compared to the freely roaming rural counterpart. Since pigs and chickens inside the city walls were likely fed with household scraps (including fish), this could also have contributed to the different human δ^15^N values.

### 4.2. Breastfeeding and weaning patterns in medieval and early modern Livonia

Most fetal, perinatal, and infant individuals show elevated δ^15^Ν values compared with the adult female means. This can be observed both in Fig 4, in which babies and infants almost all scatter above the adult means, and also especially in Fig 5, in which the individuals from the fetal to the Infant 0–1 age categories lie above the adult female value lines in both urban and rural contexts. The fetuses and perinate I individuals fall around 1–2‰ above the females, while the full term babies (perinates II) exceed 2‰ from the means.

There were three statistical outliers in this dataset, two for carbon (MK-104 and MK-155) and one for nitrogen (LK-73), all among the rural samples. However, the δ^15^Ν values of several individuals are worth discussing, since they are anomalously lower or higher in respect to the rest of the non-adult sample even if they are not statistical outliers. Individuals MK-111 (preterm around 32 weeks, δ^15^Ν = 9.57‰) and MK-74 (term baby around 39 weeks, δ^15^Ν = 10.36‰) show the lowest δ^15^Ν values of the whole sample, comparable to the rural female means (Fig 4A). According to their estimated age, it appears unlikely that they lived long enough to be breastfed, and, even in the case of age underestimation due to physiological stress, that MK-74 had enough time to develop a breastfeeding signal. We can hypothesize that these babies were reflecting in-utero values of their pregnant mothers, who may have had lower δ^15^Ν values than the rural female means [10]. Both perinates also displayed endocranial lesions and abnormal cranial porosity consistent with possible infectious or metabolic disease. In particular, MK-111 displayed porotic lesions of the orbital roofs and worm-like, flat isolated layers of new bone production with smooth appearance and vascular imprinting on the frontal bones, as well as abnormal porosity of the scapulae and ribs. MK-74 showed abnormal porosity of the ectocranial surface of the parietal bones, greater wings of the sphenoid bones, temporal bone squamae, and anterior surface of the maxilla, as well as endocranial lesions in the form of severe capillary defects of the frontal bones. Abnormal porosity of the supraspinous and infraspinous fossa of the scapulae was also observed. Low in-utero maternal values may have obscured the expected δ^15^Ν increase caused by a pathological condition, leading to the observed isotopic results. It is also possible that the effects of disease were simply not recorded in their nitrogen values; this could have happened, for example, if the child died soon after the onset of the disease, so that isotopic effects were not manifested.

On the urban counterpart, three babies clearly stood out. Individuals JK-16, JK-17 and JK-18 (all perinates I about 31 weeks old) exhibited δ^15^Ν values around 10.80‰, much lower than the other urban perinates (Fig 4A). These babies come from the same mass grave of 6 individuals located outside St Jacob’s cemetery walls (Mass Grave II) [59]. Also, these babies may be reflecting in-utero values of their mothers, who could have been rural migrants or lower class urban dwellers with lower δ^15^Ν values. The fact that they were of similar age and buried in the same grave is intriguing, and might indicate a spatial-temporal or perhaps even social relationship between them, or a similar pathological condition occurring before death (*i*.*e*. an epidemic). These individuals have been sampled for aDNA analyses in order to investigate kinship and search for evidence of infectious neonatal diseases.

A somewhat unexpected result concerns the neonates, where the mean values are closer to the perinate I category (ca. 1–2‰) than to what is expected in a nursing context. However, there are only four neonates in this sample and they show quite varied values, which complicates the task of inferring possible patterns and influences their mean values. In particular, individual JK-50 (around 42 weeks) shows quite low δ^15^Ν (11.20‰), while the other three lie within 1sd from the female means (Fig 4A). Since the estimated age seems to indicate death after birth, a reasonable explanation for their δ^15^Ν values could be that they were either not breastfed or did not live enough to develop a breastfeeding signal. Alternatively, they could have been nursed shortly by a mother (or wet-nurse) with very low δ^15^Ν values [10, 13, 36]. It can also be speculated that if the mother was unavailable or died shortly after delivery, the babies could have been fed with foods other than breastmilk (ruminant dairy, porridges, paps), which failed to meet their nutritional needs. In a historical period in which famines and epidemics were an exhausting constant, similar unfortunate scenarios may have been frequent.

Proceeding with older individuals, the infants between birth and 1 year of age (Infant 0–1 category, Fig 5) show an expected ca. 2–3‰ increase in δ^15^Ν values. In particular, individuals JK-43 (around 6 months), JK-59 (ca. 9 months) and LK-73 (about 1 year), show the highest δ^15^Ν values in the whole dataset (respectively 16.05‰, 14.86‰, and 15.63‰) (Fig 4B). This may be linked to the effects of metabolic disease: JK-43 displayed endocranial lesions, abnormal porosity of cranial and postcranial elements, and subperiosteal bone production of the skull (particularly concerning the orbital roofs), a pattern consistent with scurvy [101, 102]. For infants JK-59 and LK-73, we can also consider the effects of a systemic metabolic disease, since endocranial lesions and abnormal porosity of skull/postcranium were recorded in the former, while subperiosteal bone production and abnormal porosity of skull/postcranium were observed in the latter. For these infants we can hypothesize the effects of either severe malnutrition, or a combination of breastfeeding/weaning and metabolic disease on their isotopic values.

The results of this cross-sectional analysis seem to reflect an effective breastfeeding signal, with a successive drop between 1 and 3 years of age. The children in this category show δ^15^Ν values ranging from 12.61‰ to 13.81‰ in the urban cemetery and from 9.83‰ to 15.63‰ in the rural areas, possibly reflecting different individual choices or local customs in the weaning practice. A striking outlier is MK-155 (ca. 4 years), showing high δ^13^C and δ^15^Ν values (δ^13^C = -19.23‰, δ^15^Ν = 13.12‰) (Fig 4B). This child is a statistical outlier for stable carbon according to the IQR criterion. The individual displays bone deformation of the inferior long bones and ribs (rachitic rosary), endocranial lesions in the form of inflammatory pitting on the occipital bone squama and fiber bone deposits on the frontal and parietal bones. In addition, the child shows subperiosteal reactions on the ectocranial surface of the skull and on the tibiae, and abnormal porosity of skull and postcranium. A similar lesion pattern is consistent with a differential diagnosis of rickets [103, 104]. An additional case is represented by individual MK-104 (around 1 year of age), another statistical outlier for stable carbon (δ^13^C = -19.76‰, δ^15^Ν = 11.84‰). These values may be reflecting the dietary choices of the caregivers and, given the estimated age, perhaps the supplementary foods selected for weaning. However, the individual displayed porotic bone production on the orbits as well as abnormal porosity on the palate and around the infraorbital foramen. Bone production was also observed on the superior surface of the sphenoid lesser wings, on the pterygoid processes and mostly around the rotundus and ovale foramina. The individual also showed signs of endocranial lesions in the form of patchy capillary defects on the frontal bones above the orbital floor and on the greater wings of the sphenoid, as well as hair-on-end lesions on the temporal bone squama and inflammatory pitting on the occipital squama. Abnormal porosity of the postcranium was also detected. This pattern is compatible with a case of scurvy [101, 102]. It is worth reporting how these two cases of systemic metabolic disease show very high δ^13^C, which in MK-155 is also associated with high δ^15^N. These recorded values therefore likely reflect physiological stress. Given the estimated age, the chosen weaning strategy may have played a role in the development or worsening of a systemic metabolic disease.

The high variability encountered in this study may be explained by the historical context and social composition of this dataset, which included townspeople from different social statuses, foreign elites, and countryside migrants. People involved in childcare, including mothers and perhaps wet-nurses, likely came from different backgrounds. Hence, in addition to rural and urban food availability, childcare practices at the local or family level also ought to be considered as possible factors of diversity. For instance, according to the few available 16^th^–17^th^ century written sources, the families of German citizens and manor owners often hired Estonian wet-nurses and nannies. The 18^th^ century publicist Peter Ernst Wilde wrote that a good wet-nurse must not be a drinker, wanton, or angry woman [105]. Hiring a wet-nurse was not only a status symbol, but also added a multicultural dimension to childcare, preserving local traditions and connecting the gaps between ethnicity and social class [53]. This trend progressively decreased during the 19^th^ century, when more German women began to breastfeed their own children [106]. It is unknown whether the analyzed babies could have had access to wet-nursing, given that the sample mainly included lower status dwellers and rural communities. However, the different food habits of these people with multi-ethnic origins and personal traditions are likely to be reflected in the variability of this dataset.

Our results seem to place the duration of breastfeeding between 1 and 2 years, with the introduction of supplementary foods apparently starting around 1 year and ending close to or after 3 years of age. This is in line with isotopic data from other medieval/early modern contexts in Latvia and Lithuania [27–29]. For instance, Whitmore and colleagues [28] reported the SIA of adult individuals from the non-urban town cemetery of Alytus (Southern Lithuania, 14^th^ to early 18^th^ centuries). The sampling of adult bone and dentine formed during childhood revealed that individuals had higher δ^15^N values during the first years of life as compared to their last years of life. The authors interpreted this as a result of an incorporated breastfeeding signature, and/or of an access to terrestrial or aquatic protein sources even at very young ages at Alytus. Skipitytė et al. [29] performed cross-sectional SIA on adult and non-adult remains from three coastal communities (14^th^–early 20^th^ centuries) located near the Curonian Lagoon and the Baltic Sea in Lithuania. Their results are comparable to those of this study, and the paper reported that δ^15^N values were slightly elevated in non-adults as compared to adults, probably indicating nursing. The authors argue that breastfeeding was practiced until the age of 3–4 years, suggesting that early weaning was not the cause of death in these populations [29]. Nitrogen values comparable to this study were also reported for the children from St Gertrude church urban cemetery in Riga (Latvia, 15th–17th centuries) [107]. The results of incremental dentine SIA supported folklore and ethnographic studies reporting that Latvian children were not completely weaned until one or two years of age, and the authors attributed high δ^15^Ν values at younger ages to physiological stress in utero [107].

Observing the Infant 0–1 and Child 1–3 categories (Fig 5), a striking difference between weaning children in St Jacob’s (blue color) and in the rural areas (pink color) is seen. Urban infants and children show δ^15^Ν values respectively 1.98‰ and 1.65‰ higher compared to rural individuals. This is likely reflecting the general dietary differences discussed above, especially considering that the female baseline follows a similar trend. The women in Tartu may have had access to a wider range of foodstuffs, including imported terrestrial and marine products. This would have reasonably resulted in higher δ^15^Ν values in their breastmilk, reflected in the overall values of their nursing and weaning children. It can also be hypothesized that 1–3 year old children were breastfed longer in urban areas compared to the countryside, but this cannot be confirmed in the absence of written sources.

Unfortunately, no historical data regarding the length of breastfeeding and the age of weaning in Estonia has been recovered. Entering the realm of speculation, indirect inferences can be attempted on the basis of written sources: for instance, the 18^th^ century publicist August Wilhelm Hupel wrote that Estonian mothers let their babies crawl on the ground as soon as possible. This could have meant that lower class mothers needed their hands to participate in the housework and had to leave their babies free to crawl, but also that caregivers allowed them to be quite independent very early. Their older siblings (5–6 years old) used to look after the younger ones [108]. Hence, we could assume that 5–6 year old children should have been fully weaned, since they were able to care for their younger siblings and go to work [53]. It cannot be excluded that breastfeeding also continued when children were performing these tasks. However, it is reasonable to think that a working child could have reached a more mature status, especially considering that they were often sent far from home for apprenticeship [53]. Becoming part of the family workforce could have represented a sign of maturity for the individual, who may have been considered closer to the adult world, also from a dietary point of view.

The introduction of supplementary foods may have therefore started before one year of age, as reported for more recent contexts [106], and given that cross-sectional analyses tend to overestimate the age of weaning due to the time needed to incorporate dietary protein from non-breastmilk sources in human bone [4, 109]. Although no information exists about what kinds of foodstuffs were used to start the weaning process in medieval/early modern contexts, 19^th^–20^th^ century sources mention that barley-water, breadcrumbs in milk, and probably gruel were used as supplementary foods for infants [53, 106]. Thus, there is no reason to doubt that similar porridges and paps could have also been used in earlier times. The drop in SIA values of the children between birth and 3 years observed in this study are therefore compatible with similar mixed patterns of maternal and supplementary foods.

## 5. Conclusions

This paper reports on the first isotopic data of non-adults in Southern Estonia, providing new insights into infant diet and feeding practices in medieval and early modern Livonia. Carbon and nitrogen stable isotope analyses were performed with a cross-sectional approach, allowing reconstruction of the staple diet, detection of dietary differences between urban and rural contexts, and identification of breastfeeding and weaning patterns.

The results of this study indicate that the diet of Livonian children was mainly from terrestrial C_3_ sources and was supplemented by animal proteins. Significant differences were observed between urban and rural cemeteries, possibly linked to a wider choice of different foodstuffs (including imported marine products) accessible in urban contexts like Tartu as compared to a countryside diet with less variety of foods. A significant difference was also observed among rural subgroups, possibly due to different consumption of small amounts of C_4_ foods, but also to secular dietary changes.

Our results indicate that breastfeeding may have been practiced for 1–2 years, with the introduction of supplementary foods beginning around 1 year. The weaning process appears to have been concluded around 3 years of age, in line with isotopic data from other coeval Baltic samples. The isotopic values of older children and adolescents are comparable to the adult means, indicating that their diets were quite similar, although children may have consumed slightly fewer protein sources. This is supported by historical sources reporting that child labor began around 5–6 years of age, at which time youngsters likely began to consume an adult diet. For perinates and neonates, the isotopic values were discussed in the light of physiological stress and maternal nutrition.

This work provides a large and unique sample of well-documented material, and thus contributes to the wider picture of non-adult bioarchaeology in Eastern Europe. Despite the limitations of cross-sectional studies, initial data regarding infant dietary practices in the Eastern Baltics is presented, forming a solid baseline to understand the non-survivors; it will support future high-resolution analyses, such as incremental dentine sampling, in order to reduce the amount of speculation in the interpretation of these results. The integration of incremental data from individual survivors will allow for the creation of a finer timescale and longitudinal approach, confirming or adjusting the results presented here. Finally, future inclusion of additional individuals from other coeval rural Southern Estonian sites will contribute to obtain a more complete and homogeneous sample with fair representation of all age ranges.

## Supporting information

S1 File Statistical tests and raw isotopic data of the non-adult population from each cemetery. The file includes the results of the Shapiro-Wilk tests of normality and Mann-Whitney tests, the raw data for the carbon and nitrogen isotope analyses performed on the non-adult samples from the four urban and rural cemeteries studied, and the descriptive statistical analysis of the urban and rural female individuals categorized by age.
(DOCX)
