# Supplementary material for: Children of the grave: Investigating non-adult feeding practices in medieval and early modern Estonia through stable isotope analysis
Source: PLoS One. 2023 Jan 4;18(1):e0279546. doi: 10.1371/journal.pone.0279546 (PMC9812304; doi:10.1371/journal.pone.0279546)
Supplement: S1 File — The file includes the results of the Shapiro-Wilk tests of normality and Mann-Whitney tests, the raw data for the carbon and nitrogen isotope analyses performed on the non-adult samples from the four urban and rural cemeteries studied, and the descriptive statistical analysis of the urban and rural female individuals categorized by age. (DOCX) [file pone.0279546.s001.docx]

**Supporting Information**

**Normality Tests**

**S1 Table.** Results of the Shapiro-Wilk test of normality for non-adults from each cemetery population. The groups that do not have a normal distribution are indicated by bold font.

| **Class** | **N** | **δ^13^C W** | **δ^13^C p-value** | **δ^15^N W** | **δ^15^N p-value** |
| --- | --- | --- | --- | --- | --- |
| St Jacob | 68 | 0.984 | 0.540 | 0.973 | 0.138 |
| Lohkva | 19 | 0.965 | 0.669 | 0.930 | 0.173 |
| Mäletjärve | 2 | / | / | / | / |
| ***Makita*** | ***51*** | ***0.952*** | ***0.040*** | ***0.955*** | ***0.051*** |
| ***Total rural sample*** | ***72*** | ***0.972*** | ***0.113*** | ***0.966*** | ***0.049*** |

**S2 Table.** Results of the Shapiro-Wilk test of normality for all members of each age category. The groups that do not have a normal distribution are indicated by bold font.

| **Class** | **N** | **δ^13^C W** | **δ^13^C p-value** | **δ^15^N W** | **δ^15^N p.value** |
| --- | --- | --- | --- | --- | --- |
| 7-15 | 23 | 0.938 | 0.164 | 0.943 | 0.213 |
| ***1-7*** | ***43*** | ***0.888*** | ***0.001*** | ***0.916*** | ***0.004*** |
| Infants 0-1 | 11 | 0.945 | 0.579 | 0.899 | 0.177 |
| Neonate | 4 | 0.851 | 0.228 | 0.984 | 0.926 |
| Perinate II | 34 | 0.975 | 0.617 | 0.972 | 0.517 |
| Perinate I | 23 | 0.971 | 0.701 | 0.948 | 0.269 |
| Fetus | 2 | / | / | / | / |

**S3 Table.** Results of the Shapiro-Wilk test of normality for each age category and context. The groups that do not have a normal distribution are indicated by bold font.

| **Class** | **N** | **δ^13^C W** | **δ^13^C p-value** | **δ^15^N W** | **δ^15^N p-value** |
| --- | --- | --- | --- | --- | --- |
| Rural 7-15 | 19 | 0.943 | 0.298 | 0.955 | 0.473 |
| ***Rural 1-7*** | ***37*** | ***0.882*** | ***0.001*** | ***0.901*** | ***0.003*** |
| ***Rural Infants 0-1*** | ***8*** | ***0.882*** | ***0.197*** | ***0.722*** | ***0.004*** |
| Rural Neonate | 2 | / | / | / | / |
| Rural Perinate II | 5 | 0.967 | 0.858 | 0.945 | 0.703 |
| Rural Perinate I | 1 | / | / | / | / |
| Rural Fetus | 0 | / | / | / | / |
| ***Total rural non-adults*** | ***72*** | ***0.972*** | ***0.113*** | ***0.966*** | ***0.049*** |
| Urban 7-15 | 4 | 0.947 | 0.700 | 0.852 | 0.231 |
| Urban 1-7 | 6 | 0.832 | 0.113 | 0.874 | 0.241 |
| Urban Infants 0-1 | 3 | / | / | / | / |
| Urban Neonates | 2 | / | / | / | / |
| Urban Perinate II | 29 | 0.978 | 0.795 | 0.955 | 0.244 |
| Urban Perinate I | 22 | 0.973 | 0.768 | 0.940 | 0.201 |
| Urban Fetus | 2 | / | / | / | / |
| Total urban non-adults | 68 | 0.984 | 0.540 | 0.973 | 0.138 |

**S4 Table.** Results of the Shapiro-Wilk test of normality for the adult females. The groups that do not have a normal distribution are indicated by bold font.

| **Class** | **N** | **δ^13^C W** | **δ^13^C p-value** | **δ^15^N W** | **δ^15^N p-value** |
| --- | --- | --- | --- | --- | --- |
| Total adult females | 12 | 0.923 | 0.312 | 0.938 | 0.478 |
| Tot urban females | 6 | 0.991 | 0.991 | 0.927 | 0.557 |
| ***Tot rural females*** | 6 | ***0.749*** | ***0.020*** | 0.930 | 0.582 |

**S5 Table.** Raw data for the carbon and nitrogen isotope analyses performed on the non-adult samples from the four urban and rural cemeteries under study. Across all contexts, 9 individuals exhibited slightly lower %C and %N, but since their collagen yield and C/N ratios were optimal, they were included in the study. They are highlighted in purple in the Table. The three individuals with insufficient collagen yield and C/N ratio are indicated by red and bold font in the Table. The 33 individuals with insufficient collagen for analysis were not included here.

R = right; L = left; P1/3 = proximal third; M1/3 = medium third; D1/3 = distal third.

| **Sample ID** | **Age Category** | **Average Age** | **δ13C**  **(‰ V-PDB)** | **δ15N**  **(‰ air N2)** | **Atm % C** | **Atm % N** | **C/N ratio** | **Collagen yield** | **Bone element** |
| --- | --- | --- | --- | --- | --- | --- | --- | --- | --- |
| JK-2 | Perinate II | 38.55 w | -21.84 | 13.64 | 12.63 | 4.41 | 3.33 | 11.48 | R Ulna M1/3 |
| JK-3 | Perinate I | 32 w | -21.24 | 12.61 | 41.13 | 14.11 | 3.40 | 3.57 | L Rib |
| JK-5 | Perinate II | 39.42 w | -21.36 | 11.98 | 11.40 | 4.01 | 3.32 | 9.18 | L Ulna M1/3 |
| JK-7 | Perinate I | 35.30 w | -21.44 | 12.50 | 15.96 | 5.52 | 3.37 | 8.48 | L Ulna M1/3 |
| JK-8 | Perinate I | 36.65 w | -20.94 | 12.26 | 27.04 | 10.29 | 3.07 | 6.98 | R Radius D1/3 |
| JK-9 | Perinate II | 39.81 w | -21.17 | 13.98 | 41.98 | 14.90 | 3.29 | 3.28 | L Rib |
| JK-10 | Perinate I | 33.76 w | -20.34 | 12.85 | 43.60 | 15.89 | 3.20 | 13.59 | R Fibula |
| JK-11 | Perinate II | 37.62 w | -20.80 | 11.84 | 33.94 | 12.22 | 3.24 | 9.89 | R Radius M1/3 |
| JK-15 | Perinate II | 40 w | -21.19 | 13.73 | 18.88 | 6.67 | 3.32 | 2.40 | R Humerus M1/3 |
| JK-16 | Perinate I | 31 w | -21.23 | 10.82 | 41.95 | 15.07 | 3.25 | 8.13 | L Scapula |
| JK-17 | Perinate I | 31.87 w | -21.09 | 10.79 | 43.29 | 15.79 | 3.20 | 13.55 | R Rib |
| JK-18 | Perinate I | 31.63 w | -21.16 | 10.81 | 40.93 | 14.66 | 3.26 | 10.26 | R Ulna M1/3 |
| JK-19 | Perinate II | 37.40 w | -21.26 | 12.01 | 43.05 | 15.44 | 3.25 | 8.73 | R Fibula |
| JK-20 | Perinate I | 36.82 w | -20.94 | 13.65 | 42.04 | 14.90 | 3.29 | 4.42 | L Rib |
| JK-21 | Perinate II | 38.63 w | -21.40 | 12.04 | 28.78 | 10.52 | 3.19 | 7.08 | R Radius M1/3 |
| JK-22 | Perinate I | 34.02 w | -20.60 | 12.70 | 28.83 | 10.48 | 3.21 | 8.62 | R Radius M1/3 |
| JK-23 | Perinate II | 39.00 w | -21.67 | 12.77 | 15.88 | 5.46 | 3.39 | 6.56 | R Humerus M1/3 |
| JK-24 | Perinate I | 34.42 w | -21.28 | 12.27 | 41.24 | 14.59 | 3.30 | 2.14 | L Humerus M1/3 |
| JK-25 | Perinate I | 34.89 w | -20.39 | 13.53 | 42.19 | 14.82 | 3.32 | 5.08 | L Rib |
| JK-26 | Perinate II | 39 w | -21.74 | 12.08 | 39.97 | 14.31 | 3.26 | 5.16 | Pars Petrosa |
| JK-28 | Perinate II | 40 w | -20.93 | 13.86 | 17.88 | 6.18 | 3.38 | 6.28 | L Rib |
| JK-32 | Perinate II | 37.97 w | -20.93 | 14.31 | 41.73 | 14.77 | 3.30 | 4.88 | L Rib |
| JK-33 | Fetus | 27.95 w | -21.04 | 13.27 | 39.85 | 14.02 | 3.32 | 4.36 | L Tibia |
| JK-36 | Perinate II | 38.44 w | -20.96 | 12.45 | 42.16 | 15.39 | 3.20 | 13.25 | L Rib |
| JK-39 | Perinate II | 41 w | -20.29 | 13.77 | 42.30 | 15.11 | 3.27 | 9.28 | L Rib |
| JK-40 | Perinate II | 37.46 w | -20.46 | 12.87 | 42.17 | 15.04 | 3.27 | 14.00 | L Rib |
| JK-42 | Child 1-7 | 5 ± 1.5 y | -21.35 | 11.03 | 41.66 | 14.81 | 3.28 | 4.83 | L Rib |
| JK-43 | Infant 0-1 | 6 ± 3 m | -21.19 | 16.05 | 9.66 | 3.29 | 3.42 | 5.22 | R Rib |
| JK-44 | Perinate I | 37.13 w | -20.96 | 14.33 | 24.81 | 8.97 | 3.23 | 15.57 | R Rib |
| JK-46 | Child 1-7 | 1 y ± 4 m | -21.29 | 13.81 | 41.70 | 14.99 | 3.25 | 7.78 | L Rib |
| JK-47 | Perinate I | 36.35 w | -21.22 | 12.99 | 33.28 | 11.82 | 3.28 | 8.54 | R Rib |
| JK-50 | Neonate | 42.42 w | -20.32 | 11.20 | 38.63 | 13.65 | 3.31 | 6.72 | R Femur |
| JK-51 | Child 1-7 | 4-5 y | -22.05 | 10.18 | 42.41 | 14.99 | 3.30 | 6.34 | R Rib |
| JK-54 | Child 1-7 | 18 ± 6 m | -21.60 | 12.61 | 16.40 | 5.73 | 3.34 | 8.10 | L Rib |
| JK-55 | Child 7-15 | 8-10 y | -20.93 | 10.23 | 38.81 | 13.72 | 3.30 | 5.08 | L Rib |
| JK-56 | Child 1-7 | 5 y ± 16 m | -21.36 | 10.43 | 19.34 | 6.57 | 3.45 | 10.31 | L Rib |
| JK-59 | Infant 0-1 | 9 m ± 3 m | -20.84 | 14.87 | 33.75 | 12.12 | 3.25 | 9.84 | R Rib |
| JK-60 | Child 7-15 | 8 y ± 24 m | -21.53 | 9.96 | 32.93 | 11.62 | 3.31 | 9.94 | L Rib |
| JK-61 | Child 1-7 | 6 y ± 24 m | -22.19 | 10.92 | 42.62 | 15.24 | 3.26 | 9.85 | L Rib |
| JK-66 | Neonate | 43.67 w | -20.28 | 13.30 | 37.95 | 13.55 | 3.27 | 7.16 | L Rib |
| JK-81 | Perinate I | 36.59 w | -21.41 | 12.16 | 41.92 | 15.21 | 3.22 | 11.75 | L Rib |
| JK-151 | Child 7-15 | 11 y ± 30 m | -21.03 | 10.96 | 39.85 | 14.32 | 3.25 | 4.54 | L Rib |
| JK-448 | Child 7-15 | 10-11 y | -21.29 | 10.12 | 39.82 | 14.43 | 3.22 | 12.73 | L Rib |
| JK-12a | Perinate II | 39.85 w | -20.72 | 13.35 | 42.03 | 15.23 | 3.22 | 7.27 | R Rib |
| JK-12b | Perinate II | 39.34 w | -20.87 | 12.93 | 33.65 | 12.43 | 3.16 | 10.15 | L Fibula M1/3 |
| JK-12c | Perinate I | 35.65 w | -20.63 | 14.44 | 42.07 | 14.87 | 3.30 | 9.95 | R Rib |
| JK-12d | Perinate II | 39.20 w | -20.38 | 12.57 | 41.31 | 14.80 | 3.26 | 4.62 | R Rib |
| JK-12e | Perinate I | 35.44 w | -20.64 | 14.73 | 40.52 | 14.22 | 3.32 | 4.71 | R Fibula |
| JK-12f | Perinate II | 41.16 w | -20.83 | 13.57 | 42.56 | 15.10 | 3.29 | 4.28 | R Femur D1/3 |
| JK-13a | Perinate I | 31.27 w | -20.93 | 12.40 | 31.42 | 11.25 | 3.26 | 3.39 | R Radius M1/3 |
| JK-13c | Perinate I | 31.81 w | -21.62 | 12.13 | 20.15 | 7.21 | 3.26 | 14.20 | L Ulna P1/3 |
| JK-13d | Perinate II | > 40 w | -20.68 | 12.58 | 40.04 | 14.14 | 3.30 | 7.23 | L Rib |
| JK-13e | Fetus | 26.82 w | -21.31 | 12.17 | 40.40 | 14.53 | 3.24 | 8.47 | L Scapula |
| JK-13f | Perinate II | 38.93 w | -21.36 | 14.37 | 39.48 | 13.58 | 3.39 | 2.41 | L Tibia M1/3 |
| JK-13g | Perinate II | 40-82 w | -20.95 | 14.02 | 40.58 | 14.13 | 3.35 | 5.46 | L Rib |
| JK-13h | Perinate II | 40.73 w | -21.11 | 14.05 | 42.45 | 15.17 | 3.27 | 4.89 | L Ulna M1/3 |
| JK-13j | Perinate II | 39.12 w | -20.56 | 13.43 | 38.30 | 13.68 | 3.27 | 5.28 | R Rib |
| JK-14a | Perinate I | 31.27 w | -20.73 | 12.69 | 42.77 | 15.45 | 3.23 | 12.60 | L Humerus P1/3 |
| JK-14b | Perinate I | 34.95 w | -20.61 | 14.26 | 40.33 | 14.41 | 3.26 | 13.33 | L Rib |
| JK-14c | Perinate II | 39.96 w | -20.26 | 12.80 | 43.43 | 15.48 | 3.27 | 7.26 | L Rib |
| JK-14d | Perinate II | 39.55 w | -21.37 | 14.41 | 12.32 | 4.33 | 3.32 | 11.04 | L Humerus M1/3 |
| JK-14e | Perinate I | 31.26 w | -21.02 | 13.97 | 40.89 | 14.91 | 3.20 | 10.58 | L Tibia M1/3 |
| JK-29a | Perinate II | 40 w | -20.83 | 14.91 | 20.76 | 7.49 | 3.23 | 14.09 | L Rib |
| JK-29b | Perinate II | 39.72 w | -20.55 | 13.94 | 42.21 | 15.12 | 3.26 | 5.15 | R Tibia |
| JK-29c | Perinate II | 39.48 w | -21.14 | 13.21 | 42.03 | 14.77 | 3.32 | 10.09 | L Rib |
| JK-35a | Perinate II | 39.01 w | -21.54 | 13.73 | 41.46 | 14.71 | 3.29 | 8.14 | L Rib |
| JK-35b | Perinate I | 29.19 w | -20.95 | 12.88 | 40.05 | 14.25 | 3.28 | 3.51 | L Rib |
| JK-71b | Infant 0-1 | 6 ± 3 m | -21.76 | 13.35 | 16.37 | 5.86 | 3.26 | 7.59 | R Rib |
| LK-1 | Child 7-15 | 12 y ± 30 m | -21.07 | 9.28 | 22.74 | 8.04 | 3.30 | 5.64 | L Rib |
| LK-3b | Child 7-15 | 7 y ± 24 m | -20.99 | 10.64 | 17.78 | 6.20 | 3.35 | 6.67 | R Rib |
| LK-11c | Child 7-15 | 8 y ± 24 m | -21.50 | 10.04 | 19.91 | 6.99 | 3.32 | 9.40 | L Rib |
| LK-15 | Child 7-15 | 7 y ± 24 m | -21.89 | 10.87 | 27.53 | 9.39 | 3.43 | 9.08 | L Rib |
| LK-16 | Child 1-7 | 2.5 - 3.5 y | -21.26 | 9.83 | 37.26 | 13.54 | 3.21 | 6.81 | L Rib |
| LK-20 | Child 7-15 | 10 y ± 2.5 y | -21.48 | 10.42 | 21.69 | 7.55 | 3.35 | 13.65 | L Rib |
| LK-22 | Child 1-7 | 2 y ± 8 m | -21.37 | 10.83 | 22.13 | 7.66 | 3.37 | 10.04 | L Rib |
| LK-28 | Perinate II | 40 w | -20.86 | 12.42 | 34.36 | 12.62 | 3.18 | 4.69 | R Radius P1/3 |
| LK-30 | Child 1-7 | 4.5 – 5.5 y | -20.79 | 8.81 | 37.56 | 13.61 | 3.22 | 10.17 | L Rib |
| LK-37 | Child 1-7 | 6 y ± 24 m | -21.27 | 9.49 | 31.39 | 11.07 | 3.31 | 2.59 | L Rib |
| LK-38 | Child 1-7 | 4 y ± 12 m | -21.05 | 10.15 | 28.94 | 10.75 | 3.14 | 7.23 | L Rib |
| **LK-41** | **Child 1-7** | **3 y ± 12 m** | **-21.12** | **12.32** | **9.74** | **3.14** | **3.61** | **0.96** | **L Rib** |
| LK-42 | Child 1-7 | 2 y ± 8 m | -21.76 | 11.99 | 34.87 | 12.30 | 3.31 | 7.53 | L Rib |
| LK-46 | Child 1-7 | 6 y ± 24 m | -20.75 | 11.11 | 22.12 | 7.61 | 3.39 | 4.10 | L Rib |
| LK-73 | Child 1-7 | 1 y ± 4 m | -20.68 | 15.63 | 23.45 | 7.83 | 3.49 | 12.14 | L Rib |
| LK-74 | Child 1-7 | 4 y ± 12 m | -22.08 | 11.71 | 26.58 | 9.48 | 3.27 | 9.41 | L Rib |
| LK-89 | Infant 0-1 | 9 m ± 3 m | -20.89 | 13.50 | 35.94 | 13.00 | 3.23 | 10.67 | R Rib |
| LK-90 | Perinate II | 38.44 w | -20.30 | 11.49 | 34.30 | 12.49 | 3.20 | 10.24 | L Radius M1/3 |
| LK-48a | Perinate II | 37.97 w | -20.93 | 11.47 | 31.54 | 11.05 | 3.33 | 9.91 | R Radius P1/3 |
| LK-48b | Perinate II | 38.94 w | -20.75 | 12.15 | 30.36 | 10.75 | 3.29 | 4.33 | R Radius M1/3 |
| MJ-39 | Child 7-15 | 8 ± 2 y | -22.42 | 11.61 | 9.43 | 3.16 | 3.48 | 12.91 | L Rib |
| MJ-40 | Child 7-15 | 10 y ± 2.5 y | -21.66 | 9.94 | 18.33 | 6.67 | 3.19 | 10.74 | L Rib |
| **MK-1** | **Child 7-15** | **9 y ± 24 m** | **-21.89** | **9.90** | **8.02** | **2.29** | **4.09** | **11.01** | **L Rib** |
| MK-3 | Child 1-7 | 18 ± 6 m | -21.04 | 11.58 | 24.52 | 8.61 | 3.33 | 1.37 | L Rib |
| MK-5 | Child 7-15 | 7 y ± 24 m | -21.51 | 8.50 | 35.09 | 12.20 | 3.36 | 3.41 | L Rib |
| MK-7 | Child 1-7 | 4 ± 1 y | -21.03 | 10.45 | 23.49 | 8.25 | 3.32 | 5.86 | L Rib |
| MK-12 | Child 1-7 | 3 ± 1 y | -20.56 | 10.04 | 40.28 | 14.04 | 3.35 | 6.70 | L Rib |
| MK-13 | Child 1-7 | 3 ± 1 y | -21.26 | 9.96 | 39.44 | 14.29 | 3.22 | 3.90 | L Rib |
| MK-15 | Child 7-15 | 10 y ± 2.5 y | -20.69 | 7.72 | 40.14 | 14.70 | 3.19 | 11.16 | L Rib |
| MK-17 | Child 1-7 | 3 y ± 12 m | -21.34 | 10.56 | 22.80 | 8.26 | 3.22 | 2.75 | R Clavicle |
| MK-19 | Child 7-15 | 11 y ± 2.5 y | -21.71 | 10.93 | 19.39 | 6.63 | 3.41 | 12.21 | L Rib |
| MK-20 | Child 1-7 | 3 ± 1 y | -21.24 | 8.99 | 39.20 | 13.89 | 3.29 | 4.89 | L Rib |
| MK-21 | Infant 0-1 | 6 m ± 3 m | -20.66 | 13.35 | 35.66 | 11.90 | 3.49 | 6.79 | R Rib |
| MK-31 | Child 1-7 | 18 ± 6 m | -20.78 | 10.59 | 36.85 | 13.05 | 3.29 | 6.09 | L Rib |
| MK-34 | Child 1-7 | 1 y ± 4 m | -21.49 | 13.82 | 20.77 | 6.94 | 3.49 | 7.85 | R Rib |
| MK-35 | Child 7-15 | 11 y ± 2.5 y | -21.28 | 10.56 | 22.95 | 8.20 | 3.25 | 8.73 | L Rib |
| MK-37 | Child 1-7 | 7 ± 2 y | -21.25 | 9.41 | 34.22 | 11.96 | 3.34 | 3.75 | L Maxilla |
| MK-38 | Neonate | 43.25 w | -20.91 | 12.03 | 19.94 | 6.93 | 3.36 | 3.40 | R Rib |
| MK-45 | Child 7-15 | 7 ± 2 y | -21.50 | 10.49 | 11.44 | 4.09 | 3.27 | 4.54 | R Rib |
| MK-50 | Child 1-7 | 6 ± 2 y | -21.21 | 9.69 | 33.97 | 11.90 | 3.33 | 3.36 | L Rib |
| MK-54 | Child 1-7 | 3 y ± 12 m | -20.80 | 11.53 | 39.14 | 14.23 | 3.21 | 9.56 | L Rib |
| **MK-58** | **Child 1-7** | **6 y ± 24 m** | **-22.40** | **10.42** | **6.14** | **1.89** | **3.78** | **6.40** | **L Rib** |
| MK-59 | Child 1-7 | 2 y ± 8 m | -21.15 | 11.41 | 40.90 | 14.18 | 3.36 | 4.23 | L Rib |
| MK-74 | Perinate II | 38.45 w | -21.34 | 10.63 | 13.82 | 4.73 | 3.41 | 6.35 | L Rib |
| MK-75 | Infant 0-1 | 9 m ± 3 m | -20.45 | 13.52 | 29.15 | 10.19 | 3.34 | 6.83 | L Rib |
| MK-82 | Infant 0-1 | 9 m ± 3 m | -22.40 | 10.39 | 19.24 | 6.84 | 3.28 | 2.16 | R Rib |
| MK-84 | Infant 0-1 | 9 m ± 3 m | -21.71 | 12.66 | 15.90 | 5.27 | 3.52 | 9.04 | L Rib |
| MK-86 | Child 7-15 | 10 y ± 2.5 y | -20.76 | 9.86 | 41.79 | 14.93 | 3.27 | 10.20 | L Rib |
| MK-93 | Child 1-7 | 3 y ± 12 m | -21.49 | 8.89 | 20.85 | 6.97 | 3.49 | 10.19 | R Rib |
| MK-94 | Child 7-15 | 12 y ± 30 m | -21.13 | 9.86 | 34.09 | 12.00 | 3.31 | 3.16 | L Rib |
| MK-95 | Child 1-7 | 3 y ± 12 m | -21.50 | 11.35 | 34.03 | 11.82 | 3.36 | 1.86 | R Rib |
| MK-96 | Child 1-7 | 3 y ± 12 m | -21.30 | 9.32 | 35.40 | 12.02 | 3.44 | 3.82 | L Rib |
| MK-98 | Infant 0-1 | 6 ± 3 m | -19.99 | 12.64 | 41.51 | 14.27 | 3.39 | 11.26 | L Rib |
| MK-100 | Child 1-7 | 18 ± 6 m | -21.46 | 10.09 | 26.32 | 8.85 | 3.47 | 9.17 | L Rib |
| MK-101 | Child 1-7 | 6 y ± 24 m | -21.14 | 9.40 | 22.13 | 7.65 | 3.38 | 9.74 | L Rib |
| MK-104 | Child 1-7 | 1 y ± 4 m | -19.76 | 11.84 | 41.93 | 15.06 | 3.25 | 6.85 | L Rib |
| MK-108 | Infant 0-1 | 6 ± 3 m | -20.09 | 12.78 | 33.24 | 11.29 | 3.44 | 3.83 | R Rib |
| MK-111 | Perinate I | 31.91 w | -21.51 | 9.57 | 37.95 | 13.14 | 3.37 | 2.22 | L Humerus M1/3 |
| MK-114 | Child 1-7 | 3 y ± 12 m | -22.01 | 11.15 | 28.31 | 9.50 | 3.47 | 10.33 | L Rib |
| MK-116 | Child 7-15 | 15 y ± 36 m | -20.97 | 10.33 | 41.58 | 14.94 | 3.25 | 11.48 | L Rib |
| MK-117 | Child 1-7 | 3 y ± 12 m | -21.58 | 9.13 | 39.33 | 14.01 | 3.27 | 4.90 | R Rib |
| MK-122 | Child 7-15 | 9 y ± 24 m | -21.19 | 9.63 | 37.42 | 13.20 | 3.31 | 7.72 | L Rib |
| MK-123 | Child 7-15 | 7 y ± 24 m | -20.81 | 10.38 | 41.49 | 14.78 | 3.28 | 5.02 | L Rib |
| MK-125 | Child 1-7 | 2 y ± 8 m | -21.07 | 10.77 | 25.11 | 8.85 | 3.31 | 8.48 | L Rib |
| MK-129 | Infant 0-1 | 1 y ± 4 m | -20.38 | 13.35 | 36.13 | 12.83 | 3.28 | 3.49 | R Rib |
| MK-130 | Neonate | > 40 w | -20.49 | 12.74 | 37.07 | 13.29 | 3.25 | 4.44 | L Rib |
| MK-133 | Child 1-7 | 2.5-3.5 y | -21.36 | 10.35 | 34.59 | 12.16 | 3.32 | 3.29 | L Rib |
| MK-136 | Child 7-15 | 7 y ± 24 m | -21.14 | 8.61 | 39.63 | 14.09 | 3.28 | 7.42 | L Rib |
| MK-145 | Child 1-7 | 3 y ± 12 m | -21.83 | 9.92 | 41.25 | 14.63 | 3.29 | 6.58 | L Rib |
| MK-149 | Child 1-7 | 3 y ± 12 m | -21.56 | 9.37 | 18.60 | 6.18 | 3.51 | 1.38 | L Rib |
| MK-152 | Child 1-7 | 4 y ± 12 m | -21.19 | 9.52 | 30.07 | 10.25 | 3.43 | 1.72 | L Rib |
| MK-155 | Child 1-7 | 4 y ± 12 m | -19.23 | 13.12 | 32.80 | 11.55 | 3.31 | 5.53 | L Rib |
| MK-156 | Child 7-15 | 8 y ± 24 m | -21.00 | 9.43 | 40.71 | 14.58 | 3.26 | 5.69 | L Rib |
| MK-71(1) | Child 1-7 | 5 y ± 16 m | -22.10 | 10.75 | 8.70 | 2.84 | 3.57 | 6.19 | L Rib |
| MK-71(2) | Child 1-7 | 3 y ± 12 m | -21.42 | 12.84 | 9.67 | 3.14 | 3.59 | 3.84 | L Rib |

**Comparative tests**

1. **Kruskal-Wallis test for equal medians for urban/rural non-adults**

**δ^13^C**

N = 140

H (chi2): 6.038

Hc (tie corrected): 6.038

p-value (same): 0.1098

There is no significant difference between sample medians.

**S6 Table.** Mann-Whitney tests between δ^13^C values of non-adults from each cemetery subgroup. Significant values in bold font. Raw p-values, uncorrected significance.

|  | **Makita** | **St Jacob** | **Lohkva** | **Mäletjärve** |
| --- | --- | --- | --- | --- |
| **Makita** |  | 0.2494 | 0.7264 | **0.03779** |
| **St Jacob** | 0.2494 |  | 0.5896 | **0.03016** |
| **Lohkva** | 0.7264 | 0.5896 |  | 0.06331 |
| **Mäletjärve** | **0.03779** | **0.03016** | 0.06331 |  |

**δ^15^N**

N = 140

H (chi2): 49.11

Hc (tie corrected): 49.11

p-value (same): 1.238e^-10^

There is a significant difference between sample medians.

**S7 Table.** Mann-Whitney tests between δ^15^N values of non-adults from each cemetery subgroup. Significant values in bold font. Raw p-values, uncorrected significance.

|  | **Makita** | **St Jacob** | **Lohkva** | **Mäletjärve** |
| --- | --- | --- | --- | --- |
| **Makita** |  | **8.616E-11** | 0.2788 | 0.7974 |
| **St Jacob** | **8.616E-11** |  | **3.244E-05** | 0.0504 |
| **Lohkva** | 0.2788 | **3.244E-05** |  | 0.8574 |
| **Mäletjärve** | 0.7974 | 0.0504 | 0.8574 |  |

**S8 Table.** Descriptive statistics of the urban and rural female individuals by age category. The individuals used here as references are part of a separate paper concerned with the adult population (Morrone, original data).

|  | | **St Jacob’s urban cemetery** | | | | | | | |
| --- | --- | --- | --- | --- | --- | --- | --- | --- | --- |
|  | **N samples** | **Mean δ^13^C ‰** | **Min** | **Max** | **SD** | **Mean δ^15^N ‰** | **Min** | **Max** | **SD** |
| **Total females** | 6 | -20.02 | -21.47 | -20.48 | 0.29 | 11.18 | 10.01 | 12.67 | 0.80 |
| **20-35** | 2 | -20.76 | -21.95 | -20.48 | 0.21 | 11.09 | 10.62 | 11.43 | 0.34 |
| **35-50** | 3 | -21.11 | -21.47 | -20.65 | 0.27 | 11.05 | 10.01 | 12.67 | 0.80 |
| **Mature adult** | 1 | -20.99 | -20.99 | -20.99 | 0.00 | 12.63 | 12.63 | 12.63 | 0.00 |
|  | | **Rural cemeteries** | | | | | | | |
| **Total females** | 6 | -21.66 | -22.39 | -20.63 | 0.45 | 11.17 | 10.39 | 12.31 | 0.63 |
| **20-35** | 2 | -21.15 | -21.66 | -20.63 | 0.51 | 10.49 | 10.39 | 10.58 | 0.09 |
| **35-50** | 1 | -21.80 | -21.80 | -21.80 | 0.00 | 11.76 | 11.76 | 11.76 | 0.00 |
| **Mature adult** | 3 | -21.79 | -21.39 | -21.21 | 0.35 | 11.28 | 10.59 | 12.31 | 0.60 |


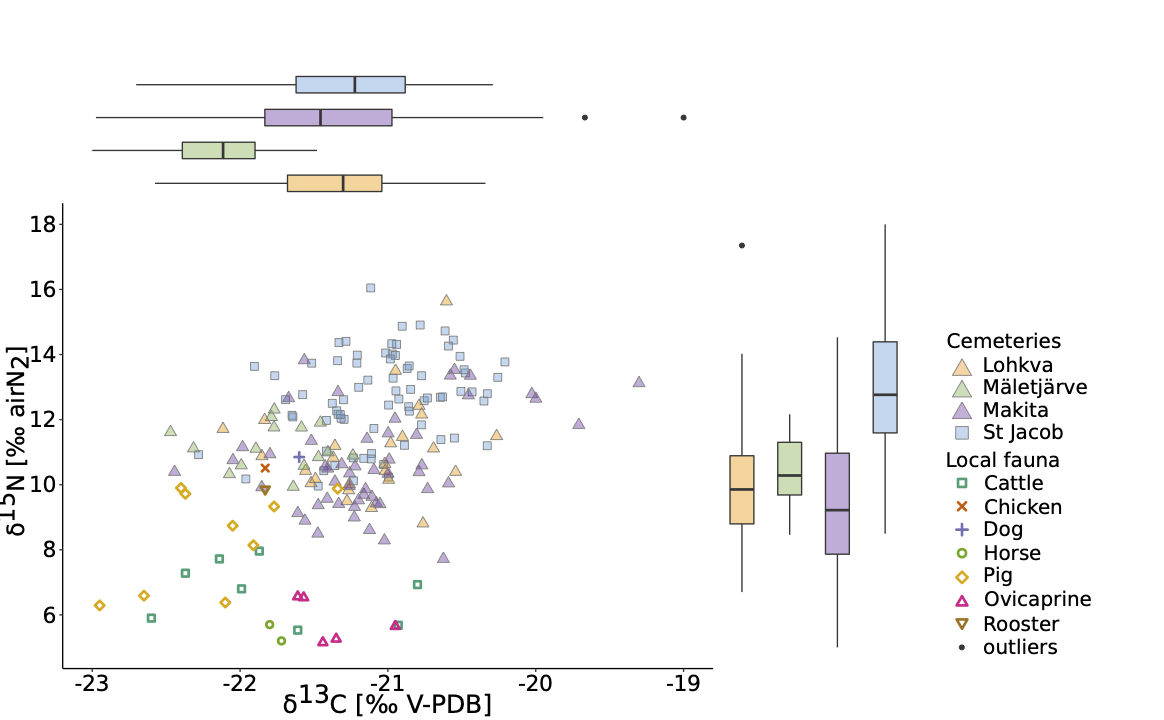


**S1 Fig.** Overview of the isotopic analysis highlighting the sites and urban-rural relationship in the entire population studied, including adults and juveniles (which are subjects of a separated paper). The scatter plot presents the δ^13^C and δ^15^N values of the samples for each cemetery along with local and coeval faunal data from Estonia (Aguraiuja-Lätti, original data; Malve, original data).
